# A General Tensorial Formulation of Acoustoelasticity and Its Representation in Cylindrical Coordinates

**DOI:** 10.3390/s26103218

**Published:** 2026-05-19

**Authors:** Yongjiang Ma, Chunguang Xu, Shuangxu Yang, Changhong Chen

**Affiliations:** 1 School of Mechanical Engineering, Beijing Institute of Technology, Beijing 100081, China; 3120215244@bit.edu.cn (Y.M.); 3220235061@bit.edu.cn (S.Y.); 3220225079@bit.edu.cn (C.C.); 2 Key Laboratory of Fundamental Science for Advanced Machining, Beijing Institute of Technology, Beijing 100081, China

**Keywords:** acoustoelasticity, ultrasonic sensing, stress measurement, tensorial equation, cylindrical coordinates

## Abstract

Acoustoelasticity provides the physical sensing principle for ultrasonic stress measurement. However, most existing formulations are restricted to isotropic media, simple stress conditions, and Cartesian coordinate systems, which limits their applicability in practical sensing scenarios involving curved and anisotropic structures. In this work, a general tensorial formulation of acoustoelasticity is developed based on the theory of incremental deformation. The proposed governing equations describe the motion of incremental displacement with explicit dependence on initial stress or strain, and are applicable to materials with arbitrary symmetry and general initial stress states. Owing to its coordinate-independent tensorial nature, the formulation can be expressed in any curvilinear coordinate system. To facilitate practical ultrasonic sensing applications, the general equations are further expanded in a cylindrical coordinate system for orthotropic materials. This enables the analysis of elastic wave propagation in curved structures such as pipelines, pressure vessels, and boreholes. The formulation establishes a direct relationship between initial stress and effective elastic properties, which determine wave velocities measurable by ultrasonic sensors, such as time-of-flight and phase velocity. The proposed approach provides a rigorous theoretical foundation for ultrasonic stress sensing and nondestructive testing, particularly for curved and anisotropic structures, and supports improved accuracy in sensor-based stress evaluation.

## 1. Introduction

The speeds of ultrasonic waves can be influenced by the initial stresses present in a medium, a phenomenon known as acoustoelasticity [1,2]. This effect provides the physical sensing principle for ultrasonic stress measurement, as it establishes a direct relationship between stress and measurable wave characteristics such as wave velocity, time-of-flight, and phase shift [3]. Ultrasonic sensing techniques based on the acoustoelastic effect are widely used in nondestructive testing (NDT) and structural health monitoring, including applications in load-bearing components, pipelines, pressure vessels, and borehole stability evaluation [4,5]. In addition, acoustoelastic methods have been applied to a wide range of materials, including metals, composites, and masonry structures, for both uniaxial [6] and multiaxial [7] stress assessment. Another important application is the inverse problem—determining higher-order elastic moduli by applying known stress levels and measuring the resulting changes in wave velocity. These moduli serve as indicators of material degradation, such as metal fatigue [8].

Over the past decades, significant progress has been made in laying the theoretical foundation for acoustoelasticity. It is based on a continuum theory of small disturbances superimposed on an elastically deformed body, formulated by Cauchy in 1829 [9]. By adding terms involving third-order elastic moduli into the constitutive equations, the modem theory of acoustoelasticity was founded by Hughes and Kelly [10]. Toupin and Bernstein [1] considered hyperelastic materials and defined three states for ultrasonic waves in stressed media. Thurston and Brugger [2] derived expressions for the velocity and the stress derivatives in terms of Second Order Elastic Constants (SOECs) and Third Order Elastic Constants (TOECs). Pao and Sachse [11] proposed a distinct formula describing the relation between the velocity and the elastic properties of materials, which laid the basis for a convenient approach for experimental verification and engineering application [3,12]. Abiza [13] elucidated in general form the dependence of speeds of body waves on the initial stress, based on a finite deformation elasticity theory founded by Odgen [14] and Shams [15]. By this stage, the classical theory of acoustoelasticity has reached a prototype [16].

Nowadays, several studies have been conducted to further develop the acoustoelastic theory. Tang [17] proposed a novel methodology for investigating acoustoelasticity based on the relationship between second harmonics and acoustoelastic effect. Tian [18] gave an analytical case where the wave velocity has complex non-linear relation with the applied hydrostatic pressure. Kube [19] related velocity change to the initial stress only through the compliance constants by a stress formulation [20] of elastic equation. Furthermore, there are some studies related to the acoustoelastic effect in anisotropic media. Such as empirical study of velocity variation due to residual stress in an unidirectional composite material [21], evaluation on the effect of the anisotropy generated by rolling on the acoustoelastic effect for aluminum alloy [22], and measurement of velocity variations induced by anisotropy and internal stresses in carbon fiber reinforced plastic composite with acoustic coefficients determined by a series of calibration tests [23].

However, many of these approaches are limited to specific material symmetries or experimental configurations. Castellano [24] did proposed a universal acoustoelastic relation regardless of the symmetric nature of wave-carrying material, based on an acoustoelastic model [25] developed in another nonlinear acoustics framework pioneered by Biot [26,27] which is different from the one adopted by [1,2,10] and the followers [12,15]. However, as manifested itself in the paper [24], this technique requires immersion ultrasonic tests to achieve the desired resolution of velocity change, which limits its applications.

Another limitation of existing acoustoelastic formulations lies in their predominant reliance on Cartesian coordinate systems. While this assumption is adequate for planar structures, it becomes insufficient for curved geometries commonly encountered in engineering applications, such as pipes, cylindrical shells, and boreholes. For certain applications [28], surface wave are more suitable to be expressed in the cylindrical system [29]. Based on the acoustoelastic model [25], Hoger gave an qualitative analysis of residual stress distribution on an cylinder [30], using a set of equations expressed in the cylindrical system, with limitation on the constitutive relation mentioned in their study.

Motivated by these limitations, the present work aims to develop a general acoustoelastic formulation, following the approach of Haupt [31], that is directly applicable to ultrasonic sensing in complex structures. Based on the theory of incremental deformation within the small-on-large framework, a fully tensorial and coordinate-independent formulation is derived. The governing equations describe the motion of incremental displacement with explicit dependence on initial stress or strain, and are applicable to materials with arbitrary symmetry and general stress states.

To facilitate practical sensing applications, the general formulation is further expanded explicitly in a cylindrical coordinate system for orthotropic materials. This enables the analysis of wave propagation in curved structures and provides a direct link between stress-dependent elastic properties and measurable ultrasonic signals. The proposed framework therefore offers a rigorous theoretical basis for improving the accuracy and reliability of ultrasonic stress sensing and nondestructive evaluation in curved and anisotropic structures.

## 2. Kinematics

In acoustoelastic theory, a solid body is typically subjected to a pre-existing (initial) stress arising from a finite deformation that maps the material from its natural configuration to a pre-deformed (initial) configuration. This initial stress modifies the material response to subsequent small perturbations and must therefore be incorporated into both the governing equations and constitutive relations.

To describe this two-stage deformation process—finite deformation followed by superimposed infinitesimal motion—a kinematic framework involving multiple configurations is introduced. Specifically, the natural, initial, and final configurations are defined, together with the corresponding deformation gradients that map between them.

In this section, the relevant configurations, deformation gradients, and associated kinematic measures are systematically established, providing the foundation for the subsequent acoustoelastic formulation.

### 2.1. Definition of Configurations, Position Vectors and Coordinate Systems

Let B be a continuum body composed of a set of material points P (for precise definitions see [32]). A configuration of B is a one-to-one mapping that assigns each material point to a unique position in Euclidean space. As the body deforms over time, it moves through a continuous sequence of spatial regions denoted by $\Omega_{t}$, for $t\in \left(0,\infty \right]$. The region $\Omega_{t}$ represents the configuration of B at time t.

**Figure 1:**
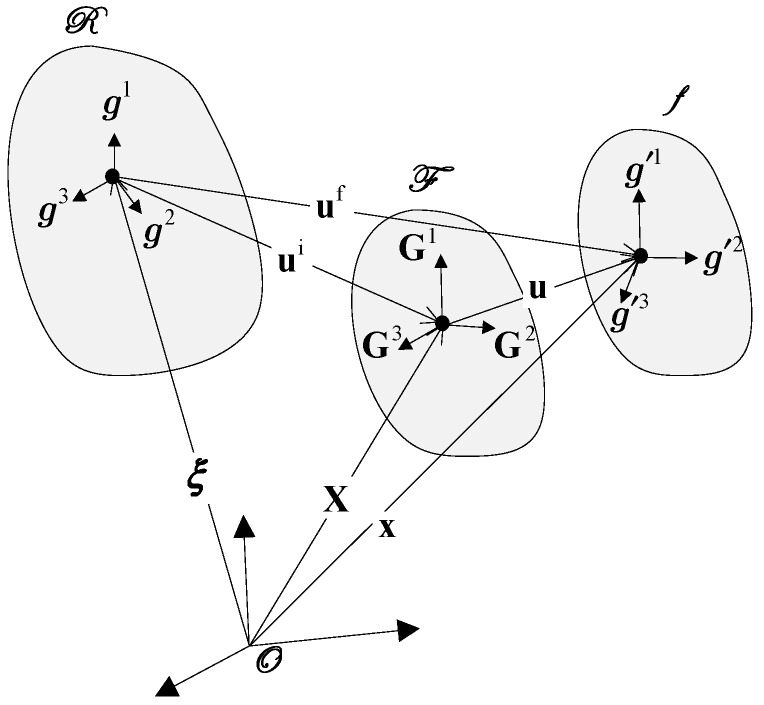
Natural, initial, and final configurations of a predeformed body.

A continuum body can undergo infinitely many configurations. In this study, we focus on three particular configurations [1], (see Figure 1):

➢ Natural configuration R: The stress-free, undeformed state.
➢ Initial configuration F: A statically deformed state with finite pre-existing stress or strain, which is the reference for incremental analysis.
➢ Final configuration f: The result of applying a dynamic perturbation, which is infinitesimal, to the pre-deformed body. It is time dependent by definition.

The position vector of a material point in each configuration is denoted by: ξ in the natural configuration; X in the initial configuration and x in the final configuration.

All position vectors are measured from the same origin in a common coordinate system. Component notation is as follows:

Greek subscripts (e.g., $\xi_{\alpha }$) refer to the natural configuration; uppercase Roman subscripts (e.g., $X_{I}$) refer to the initial configuration; lowercase Roman subscripts (e.g., $x_{i}$) refer to the final configuration.

Thus, the configurations are described via the following mappings:

(2.1a) ξ=R(P)

(2.1b) X=F(P)

(2.1c) x=f(P,t)

Since the material point P can be equivalently represented by X or ξ, the following functional dependencies arise:

(2.2a) $$X=\hat{X}(\xi )$$

(2.2b) $$x=\hat{\chi }(\xi ,t)$$

(2.2c) $$x=\hat{x}(X,t)$$

### 2.2. Definitions of Displacements

Displacement vectors quantify the relative motion of material points between different configurations. In the present formulation, three types of displacement fields are introduced, corresponding to the finite pre-deformation and the superposed incremental motion.

First, the incremental displacement vector referred to the initial configuration is defined as

(2.3a) $$U=\hat{x}(X,t)-X=U_{I}G^{I}$$

which describes the infinitesimal motion superposed on the initial configuration.

The finite displacement associated with the initial configuration may also be expressed in terms of the initial coordinates as

(2.3b) $$U^{i}=U^{i}(X)=U_{I}^{i}G^{I}$$

which represents the mapping from the natural configuration to the initial configuration expressed in the initial frame.

On the other hand, when referred to the natural configuration, the initial and final displacement vectors are defined as

(2.4a) $$u^{i}=\hat{X}(\xi )-\xi =u_{\alpha }^{i}g^{\alpha }$$

(2.4b) $$u^{f}=\hat{\chi }(\xi ,t)-\xi =u_{\alpha }^{f}g^{\alpha }$$

Hereafter, the superscripts “i” and “f” denote quantities associated with the initial and final configurations, respectively.

The incremental displacement referred to the natural configuration is then given by

(2.4c) $$u=u^{f}-u^{i}=u_{\alpha }g^{\alpha }$$

In all configurations, both covariant and contravariant basis vectors are introduced. Their definitions and properties can be found in standard textbooks on continuum mechanics such as [14,33]. The covariant and contravariant basis vectors in each configuration satisfy the duality relations

$$g^{\alpha }\cdot g_{\beta }=\delta_{\beta }^{\alpha },\;\;G^{I}\cdot G_{J}=\delta_{J}^{I},\;\;{g^{\prime }}^{i}\cdot g_{j}^{\prime }=\delta_{j}^{i}$$

where the sets $\left\{g_{\alpha },g^{\alpha }\right\}$, $\left\{G_{I},G^{I}\right\}$, and $\left\{g_{i}^{\prime },{g^{\prime }}^{i}\right\}$ correspond to the natural, initial, and current configurations, respectively.

The Euclidean inner product in the ambient space induces metric structures on each configuration through the corresponding mappings, leading to the covariant metric tensors

$$g_{\alpha \beta }=g_{\alpha }\cdot g_{\beta },\;\;G_{IJ}=G_{I}\cdot G_{J},\;\;g_{ij}^{\prime }=g_{i}^{\prime }\cdot g_{j}^{\prime }$$

and the corresponding contravariant metric tensors

$$g^{\alpha \beta }=g^{\alpha }\cdot g^{\beta },\;\;G^{IJ}=G^{I}\cdot G^{J},\;\;{g^{\prime }}^{ij}={g^{\prime }}^{i}\cdot {g^{\prime }}^{j}$$

These metric tensors provide the necessary relations for raising and lowering indices in each configuration.

### 2.3. Deformation Gradients and Displacement Gradients

Deformation gradients describe the local change in configuration of a continuum body. Three deformation gradients are introduced:

(2.5a) $$F^{f}\left(x,\xi \right)=\nabla_{\xi }\hat{\chi }\left(\xi ,t\right)={\chi^{i}}_{,\alpha }g_{i}^{\prime }⊗g^{\alpha }$$

(2.5b) $$F^{i}\left(X,\xi \right)=\nabla_{\xi }\hat{X}\left(\xi \right)={X^{I}}_{,\alpha }G_{I}⊗g^{\alpha }$$

(2.5c) $$F\left(x,X\right)=\nabla_{X}\hat{x}\left(X,t\right)={x^{i}}_{,I}g_{i}^{\prime }⊗G^{I}$$

Here, $F^{f}$, $F^{i}$, and F denote the final, initial, and incremental deformation gradients, respectively. These are two-point tensors that map tangent vectors between different configurations [34]. The symbol ⊗ denotes the tensor (dyadic) product [14].

Throughout this paper, the gradient operator is defined using a right-gradient convention, i.e., differentiation is taken with respect to the reference coordinates.

Using the identity (obtained from Equations (2.2b) and (2.2c)):

$$\hat{\chi }\left(\xi ,t\right)=\hat{x}\left(\hat{X}\left(\xi \right),t\right)$$

and applying the chain rule, the multiplicative decomposition of the deformation gradient is obtained as

(2.6) $$F^{f}=FF^{i}$$

The displacement gradients can be defined as:

(2.7a) Hf=∇ξuf=∇βufgαα⊗gβ

(2.7b) Hi=∇ξui=∇βuigαα⊗gβ

(2.7c) $$H=\nabla_{X}U=\nabla_{J}U_{I}G^{I}⊗G^{J}$$

where $H^{f}$ and $H^{i}$ are the displacement gradients defined with respect to the natural configuration, while H is the incremental displacement gradient defined with respect to the initial configuration.

From the definition of the incremental displacement U (Equation (2.3a)), one has

$$\hat{x}(X,t)=X+U(X,t)$$

Taking the gradient with respect to X on both sides yields

$$\nabla_{X}\hat{x}=\nabla_{X}X+\nabla_{X}U$$

By definitions of F (Equation (2.5c)) and H (Equation (2.7c)), it follows that

(2.8a) $$F=H+I_{F}=\nabla_{X}U+I_{F}$$

Similarly, there are:

(2.8b) $$F^{i}=\nabla_{\xi }u^{i}+I_{R}=H^{i}+I_{R}$$

(2.8c) $$F^{f}=\nabla_{\xi }u^{f}+I_{R}=\nabla_{\xi }u^{i}+\nabla_{\xi }u+I_{R}$$

From the relations above, it can be seen that:

$$HF^{i}=(F-I_{F})F^{i}=F^{f}-F^{i}=H^{f}-H^{i}=\nabla_{\xi }u$$

that is,

(2.9) $$HF^{i}=\nabla_{\xi }u$$

Here, $I_{R}$ represents the identity mapping in the natural configuration and $I_{F}$ is the identity mapping in the initial configurations. In the following, the subscript of $I_{R}$ will be omitted whenever no ambiguity arises.

Since $u^{i}$ and $U^{i}$ represent the same initial displacement field expressed with respect to the natural and initial configurations, respectively, one has

(2.10) $$u^{i}(\xi )=U^{i}(\hat{X}(\xi ))$$

Taking the gradient with respect to ξ and applying the chain rule yields

$$\nabla_{\xi }u^{i}=\nabla_{\xi }[U^{i}(X(\xi ))]=\left(\nabla_{X}U^{i}\right)\left(\nabla_{\xi }\hat{X}(\xi )\right)$$

Taking into consideration Equation (2.5b), one obtains

(2.11) $$\left(\nabla_{X}U^{i}\right)F^{i}=\nabla_{\xi }u^{i}$$

### 2.4. Strain Tensors

In order to analyze the effect of initial strain on the propagation of small perturbations, it is necessary to adopt a strain measure capable of describing finite background deformations. In the present work, the Green–Lagrange (Green) strain tensor is employed, since it naturally contains the nonlinear terms of the displacement gradient and is therefore appropriate for characterizing the finite initial deformation.

Although the subsequent formulation is concerned with the linear response of small disturbances superposed on a pre-strained body, the use of a nonlinear strain measure remains essential. It ensures that, after linearization with respect to the incremental motion, the governing equations retain the correct dependence on the initial strain, which is crucial for capturing first-order acoustoelastic effects.

For the final configuration, the Green strain tensor is defined as:

(2.12a) $$E^{f}=\frac{1}{2}\left(F^{\mathrm{fT}}F^{f}-I\right)$$

For the initial configuration:

(2.12b) $$E^{i}=\frac{1}{2}\left(F^{\mathrm{iT}}F^{i}-I\right)$$

Substituting Equations (2.7b) and (2.7c) into the definition of strain leads to a decomposition involving the displacement gradients:

(2.13a) $$E^{f}=\frac{1}{2}\left(H^{f}+H^{\mathrm{fT}}+H^{\mathrm{fT}}H^{f}\right)$$

(2.13b) $$E^{i}=\frac{1}{2}\left(H^{i}+H^{\mathrm{iT}}+H^{\mathrm{iT}}H^{i}\right)$$

The incremental Green strain tensor referred to the initial configuration is defined as:

(2.14) $$\begin{array}{l} E=\frac{1}{2}\left(F^{T}F-I_{F}\right)\\ \;=\frac{1}{2}\left(H+H^{T}+H^{T}H\right) \end{array}$$

The corresponding incremental Green strain tensor referred to the natural configuration is defined by

(2.15) $$\bar{E}=E^{f}-E^{i}$$

According to Equation (2.6), these two representations are related by

(2.16) $$\bar{E}=F^{\mathrm{iT}}EF^{i}$$

Equation (2.16) shows that $\bar{E}$ is the pull-back of E from the initial configuration to the natural configuration through the initial deformation gradient $F^{i}$.

All relations presented in this section are exact, since no approximation has yet been introduced with respect to the magnitude of the incremental deformation. It should be emphasized that E and $\bar{E}$ represent the same incremental strain measure referred to different configurations, namely the initial and natural configurations, respectively. Their mathematical forms therefore differ because of the distinct reference configurations, while their physical meaning remains the same.

## 3. Incremental Governing Equations for a Finitely Deformed Body

### 3.1. Governing Equations of Motion

Following the formulation in [31], the equation of motion for the final configuration expressed in the natural coordinate system (i.e., Lagrangian description of final motion with the natural configuration as reference configuration) is given by:

(3.1a) $$\rho^{0}\frac{\partial^{2}u}{\partial t^{2}}=\nabla_{\xi }\cdot P^{f}$$

where $\rho^{0}$ denotes the mass density in the natural configuration, u is the displacement field relative to the natural state, ξ is the material coordinate, and $P^{f}$ is the first Piola–Kirchhoff (PK1)stress tensor corresponding to the final state. The properties of PK1 stress tensor are detailed in [35]. These tensors are two-point tensors, because their geometric definition involves an area element referred to one configuration and a traction force evaluated in another configuration.

Alternatively, when expressed with respect to the initial configuration (i.e., Lagrangian description of final motion with the initial configuration as reference configuration), the corresponding equation of motion reads:

(3.1b) $$\rho^{i}\frac{\partial^{2}U}{\partial t^{2}}=\nabla_{X}\cdot p^{f}$$

where $\rho^{i}$ is the density in the initial configuration, U is the displacement relative to the initial state, X is the coordinate in the initial frame, and $p^{f}$ is the corresponding PK1 stress tensor defined with respect to the initial configuration.

In parallel, the static equilibrium (or quasi-static approximation) of the initial deformation state can be described by the following equations:

(3.2a) $$\nabla_{\xi }\cdot P^{i}=0$$

(3.2b) $$\nabla_{X}\cdot \sigma^{i}=0$$

where $\sigma^{i}$ denotes the Cauchy stress tensor in the initial configuration, and $P^{i}$ is the associated PK1 stress tensor referred to the natural configuration. Equation (3.2a) is written in the Lagrangian description with the natural configuration as reference, whereas Equation (3.2b) is expressed in the Eulerian description with the initial configuration treated as the current configuration.

It should be noted that the governing equations above are formulated in different configurations and therefore involve different stress measures. In particular, Equations (3.1) and (3.2a) are expressed in terms of PK1 stresses, whereas Equations (3.2b) involves the Cauchy stress in the initial configuration.

For the purpose of constitutive modeling under finite deformation, the stress and strain measures must be energetically conjugated. Since the Green strain tensor is adopted as the strain measure, the corresponding stress must be the second Piola–Kirchhoff (PK2) stress.

Consequently, the stress measures appearing in the governing equations are not directly compatible with the chosen strain measure and must be transformed into a unified PK2 form. This requirement motivates the introduction of stress transformation relations in the following section, which enables a consistent formulation of the incremental constitutive and motion equations.

### 3.2. Stress Measures and Inter-Configuration Transformations

The PK1 stress tensors are related to the PK2 stress tensors $T^{f}$ $T^{i}$ and $\tau^{f}$, which are defined within the reference configuration, via the following relations [31]:

(3.3a) $$P^{f}\left(\xi ,x\right)=F^{f}T^{f}$$

(3.3b) $$P^{i}\left(\xi ,X\right)=F^{i}T^{i}$$

(3.3c) $$p^{f}\left(X,x\right)=F\tau^{f}$$

Here, the notation $P^{f}(\xi ,x)$ indicates that the area element is referred to the natural configuration R while the force vector is evaluated in the final configuration f. Similar interpretations apply to the other stress measures.

The corresponding first Piola–Kirchhoff and Cauchy stress tensors are related through the standard transformation formulas

(3.4a) $$\begin{array}{c} P^{i}=J^{i}\sigma^{i}{\left(F^{i}\right)}^{-T} \end{array}$$

(3.4b) $$\begin{array}{c} P^{f}=J^{f}\sigma^{f} \end{array}{\left(F^{f}\right)}^{-T}$$

(3.4c) $$\begin{array}{c} p^{f}=J\sigma^{f}F^{-T} \end{array}$$

where $\sigma^{f}$ is the Cauchy stress tensor in the final configuration and denotes the corresponding stress quantity represented in the formulation based on the initial configuration. And

$$J^{f}=F^{f},\;J^{i}=F^{i},\;J=F$$

Combining Equations (3.3a)–(3.3c) with Equations (3.4a)–(3.4c), one obtains the standard relations between Cauchy stress tensor and PK2 tensors

(3.5a) $$\begin{array}{c} \sigma^{i}=\frac{1}{J^{i}}F^{i}T^{i}{\left(F^{i}\right)}^{T} \end{array}$$

(3.5b) $$\begin{array}{c} \sigma^{f}=\frac{1}{J^{f}}F^{f}T^{f}{\left(F^{f}\right)}^{T} \end{array}$$

(3.5c) $$\begin{array}{c} \tau^{f}=JF^{-1}\sigma^{f}F^{-T} \end{array}$$

These formulas provide the stress transformations between different configurations and will be used repeatedly in the subsequent derivation of the incremental motion equations.

### 3.3. Derivation of Incremental Motion Equations

#### 3.3.1. Referred to the Natural Configuration

Rewriting Equation (3.3a), one has

$$P^{f}=F^{f}T^{f}=F^{f}\left(T^{f}-T^{i}\right)+F^{f}T^{i}$$

Using relations $F^{f}=FF^{i}$ (Equation (2.5)) this becomes

$$P^{f}=F^{f}T+FF^{i}T^{i}$$

where T is the incremental Kirchhoff stress tensor in the natural configuration, defined by:

(3.6) $$T=T^{f}-T^{i}$$

Next, using Equation (2.8a), and retaining terms up to first order in the incremental deformation, one obtains

$$P^{f}=F^{f}T+F^{i}T^{i}+HF^{i}T^{i}$$

Using $F^{i}T^{i}=P^{i}$ (Equation (3.3b)) together with $HF^{i}=\nabla_{\xi }u$ (Equation (2.9)), this becomes:

(3.7) $$P^{f}=F^{f}T+P^{i}+\left(\nabla_{\xi }u\right)\cdot T^{i}$$

Now substitute Equation (3.7) into the equation of motion in the natural system, Equation (3.1a), and subtract the equilibrium equation of the initial state, Equation (3.2a).

(3.8) $$\rho^{0}\frac{\partial^{2}u}{\partial t^{2}}=\nabla_{\xi }\cdot \left[\left(\nabla_{\xi }u^{i}+\nabla_{\xi }u+I\right)\cdot T+\left(\nabla_{\xi }u\right)\cdot T^{i}\right]$$

where Equation (2.8c) is used.

Equation (3.8) is the incremental motion equation expressed with respect to the natural configuration. It explicitly contains the initial stress measure $T^{i}$ and the initial deformation through $\nabla_{\xi }u^{i}$, and therefore already reflects the influence of finite pre-deformation on the superposed incremental motion.

#### 3.3.2. Referred to the Initial Configuration

Starting from Equation (3.3c) and using Equation (2.8a), one has

(3.9) $$p^{f}=F\tau^{f}=\tau^{f}+H\tau^{f}=\tau +\sigma^{i}+H\tau^{f}$$

where τ is the incremental PK2 stress referred to the initial configuration and is defined as

(3.10) $$\tau =\tau^{f}-\sigma^{i}$$

Although the stress tensors $\tau^{f}$ and $\sigma^{i}$ are of different types in the strict sense (being defined in different configurations), they are both expressed with respect to the initial configuration in the present formulation. Under the assumption of infinitesimal incremental deformation, the difference between their associated configurations is of higher order and can be neglected.

Therefore, for the purpose of deriving the incremental equations, these stress measures may be consistently compared and subtracted, as commonly adopted in acoustoelastic formulations (e.g., Pao [12]). This procedure can be interpreted as an identification of stress measures under a first-order approximation with respect to the incremental deformation.

Taking (3.5b) into (3.5c), one has

$$\tau^{f}=\frac{J}{J^{f}}\left(F^{-1}F^{f}\right)T^{f}\left(F^{-T}{\left(F^{f}\right)}^{T}\right)$$

And considering $F^{f}=FF^{i}$ (Equation (2.6)) and its detrimental form $J^{f}=JJ^{i}$, the equation above reduces to

(3.11) $$\begin{array}{c} \tau^{f}=\frac{1}{J^{i}}F^{i}T^{f}{\left(F^{i}\right)}^{T} \end{array}$$

Next, subtracting Equation (3.5a) from Equation (3.11) and taking into consideration (3.6) and Equation (3.10) we have

(3.12) $$\tau =\frac{1}{J^{i}}F^{i}TF^{\mathrm{iT}}$$

Subtracting Equation (3.2b) from Equation (3.1b) and substituting Equations (3.9) and (3.12), we obtain the incremental motion equation in the initial system:

(3.13) $$\rho^{i}\frac{\partial^{2}U}{\partial t^{2}}=\nabla_{X}\cdot \left(\frac{1}{J^{i}}F^{i}TF^{\mathrm{iT}}+H\tau^{f}\right)$$

For comparison, the classical elasticity equation in the natural configuration reads

(3.14) $$\rho^{0}\frac{\partial^{2}u}{\partial t^{2}}=\nabla_{\xi }\cdot T$$

Equation (3.14) is formulated in a single reference configuration and assumes infinitesimal deformation throughout. Consequently, neither initial stress nor finite pre-deformation enters the governing equation.

By contrast, Equations (3.8) and (3.13) are established in a multi-configuration framework involving the natural, initial, and final configurations. The additional terms in these equations arise from the configuration transformations associated with the finite initial deformation. In particular, the terms involving $T^{i}$, $\tau^{f}$, $F^{i}$ and H describe the coupling between the initial stress field, the finite pre-deformation, and the superposed incremental motion.

It is important to emphasize that these additional terms are introduced by geometric nonlinearity, rather than by material nonlinearity. Even when the final incremental motion is treated within a linearized regime, the presence of a finite initial deformation modifies the governing equation through the inter-configuration mappings. This is precisely the kinematic origin of the acoustoelastic effect considered in the present study.

## 4. Incremental Constitutive Relations and Explicit Acoustoelastic Formulations

### 4.1. Incremental Constitutive Relations

It is important to note that only the constitutive relation for the PK2 stress tensor T is required, since the natural configuration serves as the only objective reference configuration suitable for defining material response functions [14].

In the present study, the material is assumed to be elastic, such that the PK2 stress tensor depends solely on the Green strain tensor [36]:

$$T^{f}=g\left(E^{f}\right)$$

where g is a tensor-valued function satisfying $g\left(0\right)=0$.

To capture the influence of initial stress or strain on the propagation of small disturbances, it is necessary to expand the constitutive relation beyond the linear regime. Specifically, expanding g in a Taylor series about the natural configuration ($E^{f}=0$), one obtains

(4.1) $$\begin{array}{l} g\left(E^{f}\right)=g\left(0\right)+\frac{dg\left(0\right)}{dE^{f}}:E^{f}+\frac{1}{2!}\frac{d^{2}g\left(0\right)}{d{\left(E^{f}\right)}^{2}}::\left(E^{f}⊗E^{f}\right)\\ \;\;\;\;+\frac{1}{3!}\frac{d^{3}g\left(0\right)}{d{\left(E^{f}\right)}^{3}}:::\left(E^{f}⊗E^{f}⊗E^{f}\right)+O\left({\left\|E^{f}\right\|}^{4}\right) \end{array}$$

where “:” denotes double contraction, “::” denotes fourfold contraction, and “:::” denotes sixth-order contraction over appropriate indices.

Despite the nonlinearity of the above expansion, a second-order approximation is sufficient for acoustoelastic analysis [31], which concerns only the first-order variation in wave velocity induced by initial stress or strain. Higher-order terms would significantly increase the complexity without providing meaningful improvement for the linearized perturbation problem.

Accordingly, under the retaining rule—namely, that terms involving strain tensors of order higher than two are neglected under the assumption of small incremental deformation [10]—Equation (4.1) reduces to

(4.2) $$T^{f}=C:E^{f}+\frac{1}{2}D::\left(E^{f}⊗E^{f}\right)$$

where C denotes the second-order elastic constants, represented by a fourth-order tensor, which characterizes the linear elastic response of the material in the natural configuration. Its symmetry properties are given by

$$C_{\alpha \beta \gamma \delta }=C_{\beta \alpha \gamma \delta }=C_{\alpha \beta \delta \gamma }=C_{\gamma \delta \alpha \beta }$$

Similarly, D denotes the third-order elastic constants, represented by a sixth-order tensor, which describes the second-order (nonlinear) elastic response of the material. Its symmetry properties are

$$\begin{array}{l} D_{\alpha \beta \gamma \delta \mu \eta }=D_{\beta \alpha \gamma \delta \mu \eta }=\cdots =D_{\beta \alpha \gamma \delta \eta \mu }\\ \;\;\;=D_{\gamma \delta \alpha \beta \mu \eta }=D_{\gamma \delta \mu \eta \alpha \beta } \end{array}$$

Likewise, the PK2 stress in the initial configuration $T^{i}$ is given by:

(4.3) $$T^{i}=C:E^{i}+\frac{1}{2}D::\left(E^{i}⊗E^{i}\right)$$

Taking the difference between Equations (4.3) and (4.2), the incremental PK2 stress becomes:

$$\begin{array}{l} T=T^{f}-T^{i}\\ \;=C:\left(E^{f}-E^{i}\right)+\frac{1}{2}D::\left(E^{f}⊗E^{f}-E^{i}⊗E^{i}\right) \end{array}$$

The first term simplifies to $C:\bar{E}$, via Equation (2.16). The second term involves a quadratic strain difference, which can be expressed as:

(4.4) $$E^{f}⊗E^{f}-E^{i}⊗E^{i}=2E^{i}⊗\bar{E}+\bar{E}⊗\bar{E}$$

Under the standard acoustoelastic assumption, where only first-order effects of the initial deformation are retained, the second term $\bar{E}⊗\bar{E}$ can be neglected. However, the interproduct term $2E^{i}⊗\bar{E}$ is preserved to fully capture the influence of initial stress on incremental motion [37]. It is emphasized that the linearization is performed with respect to the incremental strain $\bar{E}$, rather than the initial strain $E^{i}$. In the present small-on-large framework, $E^{i}$ may be finite, whereas $\bar{E}$ is assumed infinitesimal. Accordingly, all terms linear in $\bar{E}$ are retained, including mixed terms involving $E^{i}⊗\bar{E}$, while higher-order terms $O({\bar{E}}^{2})$ are neglected.

Based on the above considerations, the linearized constitutive relation for the incremental stress can be written as

(4.5) $$T=C:\bar{E}+D::\left(E^{i}⊗\bar{E}\right)$$

To further simplify the formulation and highlight the leading-order contribution, the incremental Green strain tensor is expanded as

$$\bar{E}=e+R+O\left(\varepsilon^{2}\right)$$

where e is the infinitesimal strain tensor defined by

$$e=\frac{1}{2}\left[\nabla_{\xi }u+{\left(\nabla_{\xi }u\right)}^{T}\right]$$

and R denotes the interaction term

(4.6) $$R=\frac{1}{2}\left[{\left(\nabla_{\xi }u\right)}^{T}\left(\nabla_{\xi }u^{i}\right)+{\left(\nabla_{\xi }u^{i}\right)}^{T}\left(\nabla_{\xi }u\right)\right]$$

with ε=|H| representing the magnitude of the incremental displacement gradient.

Substituting this expansion into Equation (4.5), one obtains

(4.7a) $$T=C:\left(e+R\right)+D::\left(e^{i}⊗e\right)$$

where terms of order higher than O(ε) have been neglected.

It is noted that the above constitutive relation is linear in the incremental displacement gradient, i.e., T depends linearly on $\nabla_{\xi }u$. The initial field quantities act as parametric coefficients, thereby modulating the incremental response without altering its linear structure.

Finally, using the symmetry properties of C and D, the component form of Equation (4.7a) can be expressed as

(4.7b) $$T^{\alpha \beta }=C^{\alpha \beta \gamma \delta }\nabla_{\gamma }u_{\delta }+C^{\alpha \beta \rho \delta }g^{\sigma \gamma }\nabla_{\sigma }{u^{i}}_{\rho }\nabla_{\gamma }u_{\delta }+D^{\alpha \beta \gamma \delta \varepsilon \eta }\nabla_{\gamma }{u^{i}}_{\delta }\nabla_{\varepsilon }u_{\eta }$$

### 4.2. Acoustoelastic Formulation in the Natural Configuration

By substituting Equations (4.7a) and (4.6) into Equations (3.8), the incremental equation of motion with the constitutive relation fully expressed in the natural coordinate system is obtained as

(4.8) $$\rho^{0}\frac{\partial^{2}u}{\partial t^{2}}=\nabla_{\xi }\cdot \left\{\left(\nabla_{\xi }u^{i}+\nabla_{\xi }u+I\right)\cdot \left[C:\left(e+R\right)+D::\left(e^{i}⊗e\right)\right]+\left(\nabla_{\xi }u\right)\cdot T^{i}\right\}$$

Applying the standard simplification rule of acoustoelasticity—namely, neglecting higher-order infinitesimal terms in the strain tensors—the above equation reduces to

(4.9) $$\rho^{0}\frac{\partial^{2}u}{\partial t^{2}}=\nabla_{\xi }\cdot \left[C:e+C:R+\left(\nabla_{\xi }u^{i}\right)\cdot \left(C:e\right)+D::\left(e^{i}⊗e\right)+\left(\nabla_{\xi }u\right)\cdot T^{i}\right]$$

This reduction is justified by the following order-of-magnitude estimates:

$$\begin{array}{l} \left[C:\left(e+R\right)\right]\nabla_{\xi }u\sim O\left(\varepsilon^{2}\right)\\ \left(C:R\right)\nabla_{\xi }u^{i}\sim O\left(\varepsilon^{i2}\right)\\ \left[D::\left(e^{i}⊗e\right)\right]\left(\nabla_{\xi }u^{i}+\nabla_{\xi }u\right)\sim O\left(\varepsilon^{2}\right)+O\left(\varepsilon^{i2}\right) \end{array}$$

where the symbol “~” denotes “of the order of”, and $\varepsilon^{i}=|H^{i}|$ represents the magnitude of the initial deformation gradient.

The component form of Equation (4.9) can be written as

(4.10) $$\rho^{0}\frac{\partial^{2}u_{\beta }}{\partial t^{2}}=\nabla_{\alpha }\left(\Lambda^{\alpha \beta \gamma \delta }\nabla_{\gamma }u_{\delta }+g^{\alpha \mu }g^{\gamma \eta }g^{\beta \delta }{T^{i}}_{\mu \eta }\nabla_{\gamma }u_{\delta }\right)$$

The equivalent elasticity tensor $\Lambda^{\alpha \beta \gamma \delta }$ is defined by:

(4.11a) $$\Lambda^{\alpha \beta \gamma \delta }=C^{\alpha \beta \gamma \delta }+C^{\alpha \beta \rho \delta }g^{\sigma \gamma }\nabla_{\sigma }{u^{i}}_{\rho }+C^{\alpha \sigma \gamma \delta }g^{\rho \beta }\nabla_{\sigma }{u^{i}}_{\rho }+D^{\alpha \beta \gamma \delta \varepsilon \eta }\nabla_{\varepsilon }{u^{i}}_{\eta }$$

Here, $\Lambda^{\alpha \beta \gamma \delta }$ represents the effective elasticity tensor that depends on the initial deformation. In the absence of initial deformation, it reduces to the classical elastic tensor $C^{\alpha \beta \gamma \delta }$, while the additional terms account for the influence of the initial strain field and the third-order elastic moduli on wave propagation.

For compactness, the above expression can be written in tensorial form as

(4.11b) $$\Lambda =C+P_{1243}(C\cdot \nabla_{\xi }u^{i})+P_{1342}(C\cdot \nabla_{\xi }u^{i})+D:\nabla_{\xi }u^{i}$$

where the symbol “⋅” denotes single contraction over one pair of indices, producing a fourth-order tensor from the contraction between C and $\nabla_{\xi }u^{i}$.

The operators $P_{ijkl}$ denote index permutation operators acting on the resulting fourth-order tensor, rearranging its indices according to the mapping

(α,β,γ,δ)→(i,j,k,l)

Together, Equations (4.10) and (4.11) constitute the general acoustoelastic formulation in the natural configuration (Lagrangian description with respect to the initial deformation) under an arbitrary curvilinear coordinate system. This formulation governs the propagation of small disturbances in a medium subjected to an initial stress field. The additional terms, compared to classical linear elasticity, arise from the geometric and constitutive nonlinearities associated with the initial deformation.

When specialized to a Cartesian coordinate system, the metric tensor reduces to the Kronecker delta, and the covariant derivatives reduce to partial derivatives. Upon relabeling the free indices and exploiting the symmetry of the initial stress tensor, Equations (4.10) and (4.11) reduce to:

(4.12) $$\rho^{0}\frac{\partial^{2}u_{\beta }}{\partial t^{2}}=\frac{\partial }{\partial \xi_{\alpha }}\left(\Lambda^{\alpha \beta \gamma \delta }\frac{\partial u_{\gamma }}{\partial \xi_{\delta }}+{T^{i}}_{\gamma \alpha }\frac{\partial u_{\beta }}{\partial \xi_{\gamma }}\right)$$

(4.13) $$\Lambda^{\alpha \beta \gamma \delta }=C^{\alpha \beta \gamma \delta }+C^{\alpha \beta \rho \delta }\frac{\partial {u^{i}}_{\gamma }}{\partial \xi_{\rho }}+C^{\rho \beta \gamma \delta }\frac{\partial {u^{i}}_{\alpha }}{\partial \xi_{\rho }}+D^{\alpha \beta \gamma \delta \varepsilon \eta }\frac{\partial {u^{i}}_{\eta }}{\partial \xi_{\varepsilon }}$$

It is readily verified that Equation (4.12) coincides with Equation (23) in [11], while Equation (4.13) corresponds to Equation (24), both of which are widely referenced in acoustoelastic literature [17].

For a homogeneously predeformed medium, where the initial stress tensor ${T^{i}}_{\mu \eta }$ and the elastic coefficients are spatially constant, Equation (4.10) simplifies further to:

(4.14) $$\rho^{0}\frac{\partial^{2}u_{\beta }}{\partial t^{2}}=A^{\alpha \beta \gamma \delta }\nabla_{\alpha }\nabla_{\gamma }u_{\delta }$$

where the acoustoelastic coefficient tensor is defined as

(4.15) $$A^{\alpha \beta \gamma \delta }=\Lambda^{\alpha \beta \gamma \delta }+g^{\alpha \mu }g^{\gamma \eta }g^{\beta \delta }{T^{i}}_{\mu \eta }$$

To expand the second-order covariant derivative $\nabla_{\alpha }\nabla_{\gamma }u_{\delta }$ in a general curvilinear coordinate system, we first write

$$\nabla_{\gamma }u_{\delta }=\frac{\partial u_{\delta }}{\partial \xi^{\gamma }}-\Gamma_{\gamma \delta }^{\eta }u_{\eta }$$

where $\Gamma_{\gamma \delta }^{\eta }$ denotes the Christoffel symbol [38]. Taking the covariant derivative again yields

(4.16) $$\begin{array}{l} \nabla_{\alpha }\nabla_{\gamma }u_{\delta }=\frac{\partial^{2}u_{\delta }}{\partial \xi^{\alpha }\partial \xi^{\gamma }}-\frac{\partial \Gamma_{\gamma \delta }^{\eta }}{\partial \xi^{\alpha }}u_{\eta }-\Gamma_{\gamma \delta }^{\eta }\frac{\partial u_{\eta }}{\partial \xi^{\alpha }}\\ \;\;\;\;-\Gamma_{\alpha \gamma }^{\rho }(\frac{\partial u_{\delta }}{\partial \xi^{\rho }}-\Gamma_{\rho \delta }^{\eta }u_{\eta })-\Gamma_{\alpha \delta }^{\mu }(\frac{\partial u_{\mu }}{\partial \xi^{\gamma }}-\Gamma_{\gamma \mu }^{\eta }u_{\eta }) \end{array}$$

Substituting Equation (4.16) into Equation (4.14), the fully expanded motion equation is obtained as

(4.17) $$\rho^{0}\frac{\partial^{2}u_{\beta }}{\partial t^{2}}=A^{\alpha \beta \gamma \delta }\left[\begin{array}{l} \frac{\partial^{2}u_{\delta }}{\partial \xi^{\alpha }\partial \xi^{\gamma }}-u_{\eta }\frac{\partial \Gamma_{\gamma \delta }^{\eta }}{\partial \xi^{\alpha }}-\Gamma_{\gamma \delta }^{\eta }\frac{\partial u_{\eta }}{\partial \xi^{\alpha }}\\ \;-\left(\frac{\partial u_{\mu }}{\partial \xi^{\gamma }}-u_{\eta }\Gamma_{\gamma \mu }^{\eta }\right)\Gamma_{\alpha \delta }^{\mu }-\left(\frac{\partial u_{\delta }}{\partial \xi^{\rho }}-u_{\eta }\Gamma_{\rho \delta }^{\eta }\right)\Gamma_{\alpha \gamma }^{\rho } \end{array}\right]$$

which reduces exactly to Equation (32) in [11] when specialized to the Cartesian case where all Christoffel symbols vanish.

### 4.3. Acoustoelastic Formulation in the Initial Configuration

Given that the difference between the final stress tensor $\tau^{f}$ and the initial stress $\sigma^{i}$ is of first order with respect to the incremental deformation, i.e.,

$$\tau =\tau^{f}-\sigma^{i}=O(\varepsilon )$$

where $\varepsilon =\left|H\right|$ denotes the magnitude of the incremental displacement gradient, it follows that

$$H\tau^{f}-H\sigma^{i}=H(\tau^{f}-\sigma^{i})=O(\varepsilon^{2})$$

According to the perturbation retention rule, whereby only terms up to first order in the incremental deformation are retained, contributions of order $O(\varepsilon^{2})$ can be neglected. Therefore, to first-order accuracy, Equation (3.13) can be equivalently rewritten as

(4.18) $$\rho^{i}\frac{\partial^{2}U}{\partial t^{2}}=\nabla_{X}\cdot \left(\frac{1}{J^{i}}F^{i}TF^{\mathrm{iT}}+H\sigma^{i}\right)$$

This substitution eliminates the explicit appearance of the final stress tensor, allowing the incremental motion equation to be expressed solely in terms of quantities referred to the initial configuration.

To further demonstrate that Equation (4.18) is fully expressed in the initial configuration, we substitute Equation (2.7c), yielding

(4.19) $$\rho^{i}\frac{\partial^{2}U}{\partial t^{2}}=\nabla_{X}\cdot \left(\frac{1}{J^{i}}F^{i}TF^{\mathrm{iT}}+\left(\nabla_{X}U\right)\cdot \sigma^{i}\right)$$

Among the terms on the right-hand side of Equation (4.19), the most analytically involved one is the first term inside the parentheses. To examine its structure, we temporarily omit the prefactor $1/J^{i}$ and focus on the quantity $F^{i}TF^{iT}$.

(1) Component expansion and first-order approximation

From the definition of the initial deformation gradient (Equation (2.5b)), one has

$$F^{i}={X^{I}}_{,\alpha }\;G_{I}⊗g^{\alpha },\;F^{iT}={X^{J}}_{,\beta }\;g^{\beta }⊗G_{J}$$

where

$${X^{I}}_{,\alpha }=\frac{\partial X^{I}}{\partial \xi^{\alpha }}$$

The incremental PK2 stress tensor referred to the natural configuration can be written in component form as

$$T=T^{\alpha \beta }\;g_{\alpha }⊗g_{\beta }$$

Therefore,

$$F^{i}TF^{iT}=({X^{I}}_{,\alpha }G_{I}⊗g^{\alpha })(T^{\gamma \delta }g_{\gamma }⊗g_{\delta })({X^{J}}_{,\beta }g^{\beta }⊗G_{J})$$

Using the tensor multiplication rule [38] (a⊗b)(c⊗d)=(b⋅c)a⊗d, one obtains

$$F^{i}TF^{iT}={X^{I}}_{,\alpha }{X^{J}}_{,\beta }T^{\alpha \beta }G_{I}⊗G_{J}$$

Next, ${X^{I}}_{,\alpha }$ is expanded to first order. From the relation Equation (2.8b) one has, in component form,

$${X^{I}}_{,\alpha }=g_{\alpha }^{I}+\nabla_{\alpha }u^{i\sigma }\;g_{\sigma }^{I}$$

where $g_{\alpha }^{I}=g_{\alpha }\cdot G^{I}$.

Substituting this into the previous expression yields

(4.20) $$F^{i}TF^{iT}=T^{\alpha \beta }(g_{\alpha }^{I}+\nabla_{\alpha }u^{i\sigma }g_{\sigma }^{I})(g_{\beta }^{J}+\nabla_{\beta }u^{i\lambda }g_{\lambda }^{J})G_{I}⊗G_{J}$$

Under the standard acoustoelastic assumption, only the first-order correction due to the initial displacement gradient is retained, while quadratic and higher-order terms in $\nabla_{\xi }u^{i}$ are neglected. Accordingly,

(4.21) $$F^{i}TF^{iT}\approx T^{\alpha \beta }\left(g_{\alpha }^{I}g_{\beta }^{J}+\nabla_{\alpha }u^{i\sigma }g_{\sigma }^{I}g_{\beta }^{J}+g_{\alpha }^{I}\nabla_{\beta }u^{i\lambda }g_{\lambda }^{J}\right)G_{I}⊗G_{J}$$

It should be emphasized that the neglected terms are quadratic in the initial displacement gradient, for example $\nabla_{\alpha }u^{i\sigma }\nabla_{\beta }u^{i\lambda }g_{\sigma }^{I}g_{\lambda }^{J}$ whereas all first-order terms in the initial displacement gradient are retained, since they represent the leading-order modulation of the acoustoelastic coefficients induced by the initial finite deformation.

(2) Substitution of the linearized constitutive relation

Substituting the linearized incremental constitutive relation (4.7b) into Equation (4.21), and expanding all terms while retaining only those that are linear in either the initial displacement gradient or the incremental displacement gradient, one obtains

(4.22) $$\begin{array}{l} F^{i}TF^{\mathrm{iT}}=g^{\alpha \xi }g^{\beta \lambda }\left({C_{\alpha \beta }}^{\gamma \delta }\nabla_{\gamma }u_{\delta }+{C_{\alpha \beta }}^{\gamma \delta }\nabla_{\gamma }u^{i\rho }\nabla_{\delta }u_{\rho }+{D_{\alpha \beta }}^{\gamma \delta \varepsilon \eta }\nabla_{\gamma }{u^{i}}_{\delta }\nabla_{\varepsilon }u_{\eta }\right)\\ \;\;\;\;\;\times \left(\nabla_{\xi }u^{i\sigma }g_{\sigma }^{I}g_{\lambda }^{J}+g_{\xi }^{I}\nabla_{\lambda }u^{i\sigma }g_{\sigma }^{J}+g_{\xi }^{I}g_{\lambda }^{J}\right)G_{I}⊗G_{J}\\ \;\;=\left({C_{\alpha \beta }}^{\gamma \delta }\nabla_{\gamma }u_{\delta }+{C_{\alpha \beta }}^{\gamma \delta }\nabla_{\gamma }u^{i\rho }\nabla_{\delta }u_{\rho }+{D_{\alpha \beta }}^{\gamma \delta \varepsilon \eta }\nabla_{\gamma }{u^{i}}_{\delta }\nabla_{\varepsilon }u_{\eta }\right)\\ \;\;\;\times \left(g^{\alpha \xi }\nabla_{\xi }u^{i\sigma }g_{\sigma }^{I}g^{\beta J}+g^{\alpha I}g^{\beta \lambda }\nabla_{\lambda }u^{i\sigma }g_{\sigma }^{J}+g^{\alpha I}g^{\beta J}\right)G_{I}⊗G_{J}\\ \;\;=\left(C^{IJ\gamma \delta }\nabla_{\gamma }u_{\delta }+C^{IJ\gamma \delta }\nabla_{\gamma }u^{i\rho }\nabla_{\delta }u_{\rho }+D^{IJ\gamma \delta \varepsilon \eta }\nabla_{\gamma }{u^{i}}_{\delta }\nabla_{\varepsilon }u_{\eta }\right.\\ \;\;\;\left.+g_{\sigma }^{I}C^{\xi J\gamma \delta }\nabla_{\xi }u^{i\sigma }\nabla_{\gamma }u_{\delta }+C^{I\lambda \gamma \delta }\nabla_{\lambda }u^{i\sigma }g_{\sigma }^{J}\nabla_{\gamma }u_{\delta }\right)G_{I}⊗G_{J} \end{array}$$

Two important remarks should be made. First, the last two terms in Equation (4.22) arise from the first-order corrections introduced by the left and right multiplication with the initial deformation gradient $F^{i}$, rather than from the constitutive relation itself. Second, all terms containing two factors of the initial displacement gradient (e.g., terms of the form $(\nabla u^{i})(\nabla u^{i})\nabla u$) are of second order with respect to the initial deformation and are therefore neglected under the present linearization framework.

To facilitate a unified representation in the initial configuration, the elastic constants are expressed in terms of the initial-frame components as

$$\begin{array}{c} C^{IJKL}=g_{\alpha }^{I}g_{\beta }^{J}g_{\gamma }^{K}g_{\delta }^{L}C^{\alpha \beta \gamma \delta }\\ D^{IJKLMN}=g_{\alpha }^{I}g_{\beta }^{J}g_{\gamma }^{K}g_{\delta }^{L}g_{\varepsilon }^{M}g_{\eta }^{N}D^{\alpha \beta \gamma \delta \varepsilon \eta } \end{array}$$

Accordingly, Equation (4.22) can be further rearranged entirely in terms of initial configuration indices as

(4.23) $$\begin{array}{l} F^{i}TF^{\mathrm{iT}}=\left(C^{IJKL}g_{K}^{\gamma }g_{L}^{\delta }\nabla_{\gamma }u_{\delta }\right.\\ \;\;\;\;+C^{IJML}g_{M}^{\gamma }g_{L}^{\delta }g^{\sigma \rho }\nabla_{\gamma }{u^{i}}_{\sigma }\nabla_{\delta }u_{\rho }\\ \;\;\;\;+g^{I\rho }C^{MJKL}g_{M}^{\xi }g_{K}^{\gamma }g_{L}^{\delta }\nabla_{\xi }{u^{i}}_{\rho }\nabla_{\gamma }u_{\delta }\\ \;\;\;\;+C^{IMKL}g_{K}^{\gamma }g_{L}^{\delta }g^{\rho J}g_{M}^{\lambda }\nabla_{\lambda }{u^{i}}_{\rho }\nabla_{\gamma }u_{\delta }\\ \;\;\;\;\left.+D^{IJKLMN}g_{K}^{\gamma }g_{L}^{\delta }g_{M}^{\varepsilon }g_{N}^{\eta }\nabla_{\varepsilon }{u^{i}}_{\eta }\nabla_{\gamma }u_{\delta }\right)G_{I}⊗G_{J} \end{array}$$

(3) Configuration transformation of displacement gradients

At this stage, the displacement gradients appearing in Equation (4.23) are still expressed with respect to the natural configuration. To rewrite them entirely in the initial configuration, the transformation relations for displacement gradients established previously are employed.

From the relation $HF^{i}=\nabla_{\xi }u$ [Equation (2.9)], the component form of the incremental displacement gradient reads

(4.24) $$\nabla_{\gamma }u_{\delta }={g^{Q}}_{\delta }{X^{P}}_{,\gamma }\nabla_{P}U_{Q}$$

Similarly, from $(\nabla_{X}U^{i})F^{i}=\nabla_{\xi }u^{i}$ [Equation (2.11)], one obtains

(4.25) $$\nabla_{\gamma }{u^{i}}_{\delta }={X^{P}}_{,\gamma }\nabla_{P}{U^{i}}_{Q}{g^{Q}}_{\delta }$$

Here, the two-point tensor ${X^{P}}_{,\gamma }$ admits the expansion

(4.26) $${X^{P}}_{,\gamma }=\nabla_{\gamma }{u^{i}}_{\sigma }g^{\sigma P}+g_{\gamma }^{P}$$

which characterizes the mapping between the natural and initial configurations.

Substituting Equations (4.24) and (4.25) into Equations (4.23), one finally obtains

(4.27) $$\begin{array}{l} F^{i}TF^{\mathrm{iT}}=\left(C^{IJKL}g_{K}^{\gamma }{X^{P}}_{,\gamma }\nabla_{P}U_{L}\right.\\ \;\;\;\;+C^{IJML}g_{M}^{\gamma }g_{L}^{\delta }g^{QS}{X^{P}}_{,\gamma }{X^{R}}_{,\delta }\nabla_{P}{U^{i}}_{Q}\nabla_{R}U_{S}\\ \;\;\;\;+g^{IQ}C^{MJKL}g_{M}^{\xi }g_{K}^{\gamma }{X^{P}}_{,\xi }\nabla_{P}{U^{i}}_{Q}{X^{R}}_{,\gamma }\nabla_{R}U_{L}\\ \;\;\;\;+C^{IMKL}g^{QJ}g_{K}^{\gamma }g_{M}^{\lambda }{X^{P}}_{,\lambda }{X^{R}}_{,\gamma }\nabla_{P}{U^{i}}_{Q}\nabla_{R}U_{L}\\ \;\;\;\;\left.+D^{IJKLMN}g_{K}^{\gamma }g_{M}^{\varepsilon }{X^{P}}_{,\varepsilon }\nabla_{P}{U^{i}}_{N}{X^{R}}_{,\gamma }\nabla_{R}U_{L}\right)G_{I}⊗G_{J} \end{array}$$

In the above derivation, the following contraction relations between two-point tensor components have been used:

$$g_{L}^{\delta }{g^{Q}}_{\delta }={g^{Q}}_{L},\;g^{\sigma \rho }{g^{Q}}_{\sigma }{g^{S}}_{\rho }=g^{QS}$$

(4) Order analysis of the two-point tensor ${X^{P}}_{,\gamma }$

From Equation (4.26), the two-point tensor ${X^{P}}_{,\gamma }$ consists of a zeroth-order term $g_{\gamma }^{P}$ and a first-order correction associated with the initial displacement gradient. Specifically, it can be regarded as a quantity of the form “zeroth-order + first-order perturbation with respect to the initial field”.

In the last four terms on the right-hand side of Equation (4.27), the tensor ${X^{P}}_{,\gamma }$ always appears multiplied by $\nabla U^{i}$. Therefore, substituting the first-order correction of ${X^{P}}_{,\gamma }$ into these terms would generate contributions quadratic in the initial displacement gradient. Such terms exceed the retained order of approximation in the present formulation and must be neglected. Consequently, in these terms one may safely approximate ${X^{P}}_{,\gamma }\approx g_{\gamma }^{P}$.

However, in the first term of Equation (4.27), ${X^{P}}_{,\gamma }$ does not multiply the initial displacement gradient explicitly. In this case, the product between the first-order correction of ${X^{P}}_{,\gamma }$ and the incremental displacement gradient $\nabla_{P}U_{L}$ gives rise to terms of the form “initial field × incremental field”, which are retained in the present linearized framework. Therefore, the full expression of ${X^{P}}_{,\gamma }$ must be preserved in this term, yielding

(4.28) $$\begin{array}{cc} g_{K}^{\gamma }{X^{P}}_{,\gamma }\nabla_{P}U_{L}\;\;\; & =g_{K}^{\gamma }(\nabla_{\gamma }u_{\sigma }^{i}\;g^{\sigma P}+g_{\gamma }^{P})\nabla_{P}U_{L}\\ & =(g_{K}^{\gamma }\nabla_{\gamma }u_{\sigma }^{i}\;g^{\sigma P}+g_{K}^{P})\nabla_{P}U_{L} \end{array}$$

Next, using Equation (4.25) together with the first-order approximation ${X^{P}}_{,\gamma }\approx g_{\gamma }^{P}$ in terms already containing the initial displacement gradient, one obtains

(4.29) $$\nabla_{\gamma }u_{\sigma }^{i}\approx g_{\gamma }^{M}\nabla_{M}U_{Q}^{i}\;{g^{Q}}_{\sigma }$$

Substituting this relation into Equation (4.28) gives

(4.30) $$g_{K}^{\gamma }\nabla_{\gamma }u_{\sigma }^{i}\;g^{\sigma P}=g_{K}^{\gamma }g_{\gamma }^{M}\nabla_{M}U_{Q}^{i}\;{g^{Q}}_{\sigma }g^{\sigma P}=g^{QP}\nabla_{K}U_{Q}^{i}$$

Together with the identity $g_{K}^{P}\nabla_{P}U_{L}=\nabla_{K}U_{L}$, one obtains

(4.31) $$g_{K}^{\gamma }{X^{P}}_{\gamma }\nabla_{P}U_{L}=g^{QP}\nabla_{K}{U^{i}}_{Q}\nabla_{P}U_{L}+\nabla_{K}U_{L}$$

Equation (4.31) shows that the first-order correction of ${X^{P}}_{,\gamma }$ gives rise to an additional coupling term associated with the initial displacement gradient, while its zeroth-order part recovers the standard incremental displacement gradient. It is precisely this structure that leads to an effective elasticity tensor incorporating both material constitutive constants and geometric corrections induced by the initial finite deformation.

(5) Final expression

Substituting Equation (4.32) into Equation (4.27), and replacing ${X^{P}}_{,\gamma }$ by $g_{\gamma }^{P}$ in the remaining four terms, one finally obtains

(4.32) $$\begin{array}{l} F^{i}TF^{\mathrm{iT}}=\left(C^{IJKL}\nabla_{K}U_{L}+C^{IJML}g^{PK}\nabla_{M}{U^{i}}_{P}\nabla_{K}U_{L}+C^{IJKM}g^{PL}\nabla_{M}{U^{i}}_{P}\nabla_{K}U_{L}\right.\\ \;\;\;\;+g^{IP}C^{MJKL}\nabla_{M}{U^{i}}_{P}\nabla_{K}U_{L}+C^{IMKL}g^{JP}\nabla_{M}{U^{i}}_{P}\nabla_{K}U_{L}\\ \;\;\;\;+\left.D^{IJKLMN}\nabla_{M}{U^{i}}_{N}\nabla_{K}U_{L}\right)G_{I}⊗G_{J} \end{array}$$

It is evident from Equation (4.32) that all physical quantities and their derivatives are now fully expressed in the initial configuration. Notably, the introduction of the tensor ${X^{P}}_{,\gamma }$ generates additional coupling terms, which ensures that the resulting effective elasticity tensor retains the same symmetry structure as the material elastic constants under stress-induced conditions. This important property will be discussed in detail in the following section.

Another important quantity yet to be addressed is the determinant of the deformation gradient tensor in the initial configuration, denoted as $J^{i}$, according to its definition:

$$J^{i}=\det\left(F^{i}\right)=\det\left(H^{i}+I\right)$$

where $H^{i}$ denotes the displacement gradient.

Using the standard expansion of the determinant (see, e.g., Chp 5 of [38]) for small but finite deformation gradients, one obtains

$$\begin{array}{l} \det(I+H^{i})=1+\mathrm{tr}(H^{i})+\frac{1}{2}\left[{\left(\mathrm{tr}H^{i}\right)}^{2}-\mathrm{tr}\left(H^{i2}\right)\right]\\ \;\;\;\;\;+\frac{1}{6}\left[{\left(\mathrm{tr}H^{i}\right)}^{3}-3\mathrm{tr}H^{i}\mathrm{tr}\left(H^{i2}\right)+2\mathrm{tr}\left(H^{i3}\right)\right]\\ \;\;\;\;\;+\cdots \end{array}$$

In the present work, only the first-order approximation of initial deformation is required, yielding

$$\det(I+H^{i})\approx 1+\mathrm{tr}(H^{i})$$

The displacement gradient $H^{i}$ can be decomposed into its symmetric and skew-symmetric parts:

$$H^{i}=e^{i}+\omega^{i}$$

where $e^{i}=\left(H^{i}+H^{\mathrm{iT}}\right)/2$ is the symmetric part (linear strain) and $\omega^{i}=\left(H^{i}-H^{\mathrm{iT}}\right)/2$ is the skew-symmetric part. It follows immediately that

$$\mathrm{tr}(H^{i})=\mathrm{tr}(e^{i})$$

since the skew-symmetric tensor $\omega^{i}$ has zero trace.

Therefore, we obtain the linear approximation:

(4.33) $$J^{i}=\mathrm{tr}e^{i}+1$$

Since both $J^{i}$ and $\mathrm{tr}e^{i}$ are scalar invariants, Equation (4.33) holds in any coordinate system. Moreover, recalling that $e^{i}$ is the symmetric part of the displacement gradient $\nabla_{\xi }u^{i}$, we have:

$$\mathrm{tr}e^{i}=\mathrm{tr}\left(\nabla_{\xi }u^{i}\right)$$

From Equation (4.29), the difference between $\nabla_{\xi }u^{i}$ and $\nabla_{X}U^{i}$ is of second order. Hence, Equation (4.33) can be equivalently written as:

$$J^{i}=\mathrm{tr}\left(\nabla_{X}U^{i}\right)+1$$

Under the standard acoustoelastic assumption, retaining only first-order terms in the initial displacement gradient, the Jacobian determinant $J^{i}$ and its reciprocal are approximated by

(4.34a) $$\frac{1}{J^{i}}=1-\mathrm{tr}\left(\nabla_{X}U^{i}\right)$$

or, in component form,

(4.34b) $$\frac{1}{J^{i}}=1-\nabla_{P}U^{iP}$$

This relation should be substituted into the corresponding expression for the term in the initial configuration in Equation (2.19). As noted previously, the factor $1/J^{i}$ multiplies only the term $F^{i}TF^{iT}$. Under the same first-order approximation used in the analysis of ${X^{P}}_{,\gamma }$, only its contribution to the leading term of Equation (4.32) needs to be retained.

We thus arrive at the final form of the incremental displacement equation referred to the initial configuration:

(4.35) $$\rho^{i}\frac{\partial^{2}U_{J}}{\partial t^{2}}=\nabla_{I}\left[\left(\Sigma^{IJKL}+g^{IM}g^{KN}g^{JL}{\sigma^{i}}_{MN}\right)\nabla_{K}U_{L}\right]$$

where the effective elasticity tensor $\Sigma^{IJKL}$ is given by:

(4.36a) $$\begin{array}{l} \Sigma^{IJKL}=C^{IJKL}\left(1-g^{PQ}\nabla_{P}{U^{i}}_{Q}\right)\\ \;\;\;\;+C^{IJML}g^{PK}\nabla_{M}{U^{i}}_{P}+C^{IJKM}g^{PL}\nabla_{M}{U^{i}}_{P}\\ \;\;\;\;+g^{IP}C^{MJKL}\nabla_{M}{U^{i}}_{P}+C^{IMKL}g^{JP}\nabla_{M}{U^{i}}_{P}\\ \;\;\;\;+D^{IJKLMN}\nabla_{M}{U^{i}}_{N} \end{array}$$

Equation (4.35) is the incremental equation of motion expressed entirely in the initial (pre-deformed) configuration. This form is particularly convenient for wave-propagation analysis in stressed components, since both the geometry and the boundary conditions are naturally posed on the current body shape.

The tensor $\Sigma^{IJKL}$ represents the effective incremental elasticity tensor in the initial configuration. The prefactor $C^{IJKL}(1-g^{PQ}\nabla_{P}U_{Q}^{i})$ arises from the first-order expansion of $1/J^{i}$, and therefore reflects the volumetric effect of the initial deformation. The terms proportional to $C^{\ldots }\nabla U^{i}$ originate from the geometric mapping between configurations, while the final term involving $D^{IJKLMN}$ represents the contribution of the third-order elastic constants. Together with the initial-stress term involving $\sigma_{MN}^{i}$, these contributions describe how the effective stiffness depends explicitly on the initial state of the body.

Unlike classical elasticity, where the stiffness depends only on material properties, the present formulation shows that both the initial deformation and the initial stress modify the incremental wave equation. In ultrasonic nondestructive testing applications, this governing equation leads directly to stress-dependent wave speeds, and hence to experimentally measurable acoustoelastic coefficients.

Similarly, $\Sigma^{IJKL}$ can be expressed as

(4.36b) $$\Sigma =C-\mathrm{tr}(\nabla_{X}U^{i})C+P_{1243}\;\;\left[C\cdot (\nabla_{X}U^{i})\right]+P_{1342}\;\;\left[C\cdot (\nabla_{X}U^{i})\right]+D:\left(\nabla_{X}U^{i}\right)$$

with notations consistent with those in Equation (4.11b).

Equations (4.35) and (4.36) together constitute the general formulation of acoustoelasticity in the initial configuration for arbitrary curvilinear coordinates. When specialized to a Cartesian coordinate system, the metric tensor reduces to the Kronecker delta and the covariant derivatives become ordinary partial derivatives. With appropriate relabeling of the free indices and using the symmetry of the initial stress tensor, Equations (4.35) and (4.36) simplify to:

(4.37) $$\rho^{i}\frac{\partial^{2}U_{J}}{\partial t^{2}}=\frac{\partial }{\partial X_{I}}\left[\left(\Sigma^{IJKL}+\delta_{IK}{\sigma^{i}}_{JL}\right)\frac{\partial U_{K}}{\partial X_{L}}\right]$$

(4.38) $$\begin{array}{l} \Sigma^{IJKL}=C^{IJKL}\left(1-\frac{\partial {U^{i}}_{Q}}{\partial X_{Q}}\right)+C^{IJML}\frac{{U^{i}}_{K}}{\partial X_{M}}+C^{IJKM}\frac{{U^{i}}_{L}}{\partial X_{M}}\\ \;\;\;\;+\frac{{U^{i}}_{I}}{\partial X_{M}}C^{MJKL}+C^{IMKL}\frac{{U^{i}}_{J}}{\partial X_{M}}+D^{IJKLMN}\frac{{U^{i}}_{N}}{\partial X_{M}} \end{array}$$

here, Equation (4.37) corresponds to Equation (29), and Equation (4.38) to Equation (27), in the well-known reference [11].

The Cartesian forms Equations (4.37) and (4.38) are provided for two reasons: (i) they serve as a consistency check by recovering the classical acoustoelastic equations reported in [11]; and (ii) they offer a familiar representation for readers to connect the present tensorial formulation with standard Cartesian-based NDT implementations.

For a homogeneously pre-deformed medium, the initial stress ${\sigma^{i}}_{MN}$ and elasticity tensor $\Sigma^{IJKL}$ are spatially uniform. In this case, the governing equation (4.35) simplifies to:

(4.39) $$\rho^{i}\frac{\partial^{2}U_{J}}{\partial t^{2}}=B^{IJKL}\nabla_{I}\nabla_{K}U_{L}$$

where the acoustoelastic coefficient tensor is defined by:

(4.40) $$B^{IJKL}=\Sigma^{IJKL}+g^{IM}g^{KN}g^{JL}{\sigma^{i}}_{MN}$$

To further expand the second-order derivative term in general curvilinear coordinates, recall that for a second-order tensor $H_{KL}$, the covariant derivative is given by [38]:

$$\nabla_{I}H_{KL}=\frac{\partial H_{KL}}{\partial X^{I}}-\Gamma_{IL}^{Q}H_{KQ}-\Gamma_{IK}^{R}H_{RL}$$

where $\Gamma_{IL}^{Q}$ and $\Gamma_{IL}^{Q}$ are the Christoffel symbols in the initial configuration. Since

$$H_{KL}=\nabla_{K}U_{L}=\frac{\partial U_{L}}{\partial X^{K}}-\Gamma_{KL}^{P}U_{P}$$

the full expansion of $\nabla_{I}\nabla_{K}U_{L}$ becomes:

(4.41) $$\begin{array}{l} \nabla_{I}\nabla_{K}U_{L}=\frac{\partial^{2}U_{L}}{\partial X^{I}\partial X^{K}}-\Gamma_{KL}^{P}\frac{\partial U_{P}}{\partial X^{I}}-U_{P}\frac{\partial \Gamma_{KL}^{P}}{\partial X^{I}}\\ \;\;\;\;\;-\left(\frac{\partial U_{Q}}{\partial X^{K}}-U_{P}\Gamma_{KQ}^{P}\right)\Gamma_{IL}^{Q}-\left(\frac{\partial U_{L}}{\partial X^{R}}-U_{P}\Gamma_{RL}^{P}\right)\Gamma_{IK}^{R} \end{array}$$

Therefore, the equation of motion [Equation (4.39)] in its fully expanded form reads:

(4.42) $$\rho^{i}\frac{\partial^{2}U_{J}}{\partial t^{2}}=B^{IJKL}\left[\begin{array}{l} \frac{\partial^{2}U_{L}}{\partial X^{I}\partial X^{K}}-\Gamma_{KL}^{P}\frac{\partial U_{P}}{\partial X^{I}}-U_{P}\frac{\partial \Gamma_{KL}^{P}}{\partial X^{I}}\\ \;\;\;-\left(\frac{\partial U_{Q}}{\partial X^{K}}-U_{P}\Gamma_{KQ}^{P}\right)\Gamma_{IL}^{Q}-\left(\frac{\partial U_{L}}{\partial X^{R}}-U_{P}\Gamma_{RL}^{P}\right)\Gamma_{IK}^{R} \end{array}\right]$$

It is worth noting that, in Cartesian coordinates, all Christoffel symbols vanish, and Equation (4.42) reduces to Equation (38) in [11]. In that case, the governing equations form a constant-coefficient second-order hyperbolic system. Based on this structure, together with the plane-wave assumption, explicit analytical expressions for longitudinal and transverse wave velocities can be derived, as shown in Equation (54) of [11].

However, in the present formulation, the governing equations (e.g., Equations (4.41) and (4.42)) include additional terms arising from the curvilinear coordinate system, in which Christoffel symbols introduce spatially varying coefficients and lower-order coupling terms involving both displacement and its gradients. As a result, the equations no longer reduce to a standard constant-coefficient hyperbolic system, and the classical plane-wave solution approach used in [11] is not directly applicable.

Consequently, closed-form analytical expressions similar to Equations (53) and (54) are generally not available in curvilinear coordinates. Nevertheless, the formulation still provides a rigorous description of wave propagation, where the effective elastic coefficients determine wave characteristics that can be evaluated numerically and are directly related to measurable quantities in ultrasonic sensing.

Although no higher-order terms of the displacement field explicitly appear in Equations (4.10) and (4.35), the acoustoelastic formulation remains inherently nonlinear. This nonlinearity arises from three main sources: (i) geometric nonlinearity associated with mappings between different configurations, (ii) nonlinear strain measures through the Green strain tensor, and (iii) material nonlinearity introduced by the second-order expansion of the constitutive relation and the associated third-order elastic moduli. In the present work, only the linear influence of initial stress or strain on wave propagation is retained, which is consistent with the small-on-large framework commonly adopted in acoustoelastic theory.

In summary, this section establishes a general tensorial formulation of acoustoelasticity in both natural and initial configurations. The governing equations are expressed in a coordinate-independent form and are therefore applicable to arbitrary curvilinear coordinate systems. The primary distinction between the two formulations lies in the definition of the acoustoelastic coefficient tensors, which represent stress- and deformation-dependent effective elastic moduli governing incremental wave motion. The formulation in the natural configuration provides a conceptually transparent theoretical foundation, whereas the formulation in the initial configuration is directly suited for practical wave-propagation analysis, where boundary conditions and geometry are specified on the pre-stressed body. These formulations together provide the basis for analyzing stress-dependent wave characteristics that can be measured in ultrasonic nondestructive testing and related sensing applications [39].

## 5. Acoustoelasticity Theory in the Cylindrical System

The acoustoelastic equations derived in the previous sections are formulated in a general curvilinear coordinate system and therefore remain valid for any curvilinear coordinates. In practical applications, however, orthogonal coordinate systems are most commonly used—such as cylindrical, spherical, or natural coordinates. Cylindrical coordinates are particularly suitable for analyzing wave propagation in cylindrical components or geological structures like boreholes. Spherical coordinates are more appropriate for components with spherical surfaces or for modeling wave behavior on the Earth’s surface. For curved structures such as turbine blades or shell-like components, natural coordinates offer a more convenient framework for describing ultrasonic wave propagation. While the governing acoustoelastic equations retain their general form, their specific expressions differ among coordinate systems due to variations in the Christoffel symbols $\Gamma_{ij}^{k}$ associated with the underlying coordinate curves. In this section, we present the explicit formulation of the acoustoelastic equations in cylindrical coordinates as a representative example.

### 5.1. Cylindrical Coordinate System

In an orthonormal curvilinear coordinate system, the components of the metric tensor $g_{ij}$ satisfy the following properties [40]:

(5.1) $$\begin{array}{l} g_{ij}=g_{i}\cdot g_{j}=0,\;for\;i\neq j;\\ g^{ij}=g^{i}\cdot g^{j}=0,\;for\;i\neq j;\\ g^{\underline{ii}}=1/g_{\underline{ii}};\\ g_{11}g_{22}g_{33}=g. \end{array}$$

Here, an underline beneath a repeated index (e.g., $\underline{ii}$) indicates no summation over that index. To obtain explicit partial-differential forms of the governing equations in cylindrical coordinates, we expand the covariant derivatives in terms of Christoffel symbols. The Christoffel symbols $\Gamma_{ij}^{k}$ are related to the metric tensor $g_{ij}$ by [38]:

(5.2) $$\Gamma_{ij}^{k}=\frac{1}{2}g^{kl}\left(\frac{\partial g_{jl}}{\partial x^{i}}+\frac{\partial g_{li}}{\partial x^{j}}-\frac{\partial g_{ij}}{\partial x^{l}}\right)$$

In an orthonormal curvilinear coordinate system (as assumed here), the metric tensor is diagonal and the Christoffel symbols simplify accordingly,

(5.3) $$\begin{array}{l} \Gamma_{ij}^{k}=0,\;\left(i\neq j\neq k\neq i\right)\\ \Gamma_{\underline{i}j}^{\underline{i}}=\Gamma_{j\underline{i}}^{\underline{i}}=\frac{1}{2g_{\underline{ii}}}\frac{\partial g_{\underline{ii}}}{\partial x_{j}}\\ \Gamma_{\underline{ii}}^{j}=-\frac{1}{2}g^{\underline{jj}}\frac{\partial g_{\underline{ii}}}{\partial x^{j}} \end{array}$$

More specifically, for cylindrical system [40], there is:

(5.4) $$\begin{array}{l} \Gamma_{12}^{2}=\Gamma_{21}^{2}=\frac{1}{r};\\ \Gamma_{22}^{1}=-r,\;\\ \Gamma_{jk}^{i}=0\;\;\left(\mathrm{other}\;\mathrm{cases}\right) \end{array}$$

### 5.2. Material Symmetricity

In this section, we develop the acoustoelastic formulation for orthotropic materials, which are commonly encountered in industrial applications. For such materials, the normal stresses $\sigma_{11},\sigma_{22},\sigma_{33}$ depend only on the normal strains $\varepsilon_{11},\varepsilon_{22},\varepsilon_{33}$ and are independent of shear strains $\varepsilon_{12},\varepsilon_{23},\varepsilon_{31}$. Each shear stress depends solely on its corresponding shear strain [41].

For orthotropic solids, it is convenient to express the fourth-order elasticity tensor C in Voigt notation for engineering implementation. This matrix form is merely a contracted representation of the tensorial constitutive relation and does not alter the coordinate-independent formulation. The second-order elasticity tensor C then takes the form:

(5.5) $$C=\left[\begin{array}{cccccc} C_{11} & C_{12} & C_{13} & 0 & 0 & 0\\ C_{12} & C_{22} & C_{23} & 0 & 0 & 0\\ C_{13} & C_{23} & C_{33} & 0 & 0 & 0\\ 0 & 0 & 0 & C_{44} & 0 & 0\\ 0 & 0 & 0 & 0 & C_{55} & 0\\ 0 & 0 & 0 & 0 & 0 & C_{66} \end{array}\right]$$

This is expressed in Voigt notation using the mapping: 1→11, 2→22, 3→33, 4→23, 5→13, 6→12.

In general, the third-order elasticity tensor D has 56 independent components, but only 20 are non-zero for orthotropic materials [42]. These are:

(5.6) $$\begin{array}{l} D_{111},\;D_{222},\;D_{333}\;\\ D_{144},\;D_{255},\;D_{366}\\ D_{112},\;D_{223},\;D_{133},\;D_{113},\;D_{122},\;D_{233},\\ D_{155},\;D_{244},\;D_{344},\;D_{166},\;D_{266},\;D_{355},\\ D_{123},\;D_{456}. \end{array}$$

The orthotropic assumption is adopted here because it covers a wide range of engineering materials (e.g., rolled metals and fiber-reinforced composites) while keeping the cylindrical expansion tractable.

### 5.3. Acoustoelasticity Formulation in Cylindrical System

In the present cylindrical formulation, it is assumed that the material coordinate system coincides with both the principal directions of orthotropy and the principal directions of the initial stress. Under this assumption, the material symmetry axes are aligned with the cylindrical coordinate directions, and the initial shear stress components vanish. This configuration is representative of many practical cases, such as rolled or filament-wound cylindrical structures.

In practical engineering applications, however, the material symmetry axes and principal stress directions do not necessarily coincide with the geometric coordinate system. In such cases, the governing equations include additional coupling terms arising from the misalignment, which may lead to direction-dependent wave propagation and mode conversion. These effects can significantly influence measurable quantities in ultrasonic sensing, such as wave velocity and time-of-flight, and must therefore be properly accounted for in stress evaluation.

It should be noted that such misalignment introduces additional coupling terms in the governing equations, which may lead to direction-dependent wave propagation and mode conversion. These effects can significantly influence the propagation characteristics of elastic waves and consequently affect measurable quantities in ultrasonic sensing, such as wave velocity and time-of-flight. Therefore, proper consideration of coordinate misalignment is essential for accurate stress evaluation in practical engineering applications.

The present formulation remains applicable in such situations through standard tensor transformations. Specifically, the second- and third-order elastic constants can be transformed between coordinate systems using direction cosine matrices, as expressed in Equation (5.7). This ensures that the cylindrical acoustoelastic formulation can be extended to general cases without altering its theoretical structure.

(5.7) $$\begin{array}{l} C_{mnop}=\beta_{mi}\beta_{nj}\beta_{ok}\beta_{pl}C_{ijkl}^{\prime }\\ D_{opqrst}=\beta_{oi}\beta_{pj}\beta_{qk}\beta_{rl}\beta_{sm}\beta_{tn}D_{ijklmn}^{\prime } \end{array}$$

where $\beta_{ij}$ denotes the direction cosine matrix relating the material coordinate system to the principal stress or geometric coordinate system.

Under this symmetry and configuration, for the natural configuration, only 21 independent components of the coefficient tensor $A_{\alpha \beta \gamma \delta }$ remain which are listed in the Appendix A. Substituting into Equation (4.10), the wave equations in cylindrical coordinates become:

(5.8a) $$\begin{array}{l} \rho^{0}\frac{\partial^{2}u_{r}}{\partial t^{2}}=A_{1111}\frac{\partial^{2}u_{r}}{\partial r^{2}}+\frac{A_{1212}}{r^{2}}\frac{\partial^{2}u_{r}}{\partial \theta^{2}}+A_{1313}\frac{\partial^{2}u_{r}}{\partial z^{2}}\\ \;\;\;\;+\frac{A_{1122}+A_{1221}}{r}\frac{\partial^{2}u_{\theta }}{\partial r\;\partial \theta }+\left(A_{1331}+A_{1133}\right)\frac{\partial^{2}u_{z}}{\partial r\;\partial z}\\ \;\;\;\;+\frac{A_{1111}+A_{1122}-A_{2211}}{r}\frac{\partial u_{r}}{\partial r}-\frac{A_{1212}+A_{2222}}{r^{2}}\frac{\partial u_{\theta }}{\partial \theta }\\ \;\;\;\;+\frac{A_{1133}-A_{2233}}{r}\frac{\partial u_{z}}{\partial z}-\frac{A_{2222}}{r^{2}}u_{r} \end{array}$$

(5.8b) $$\begin{array}{l} \rho^{0}\frac{\partial^{2}u_{\theta }}{\partial t^{2}}=A_{2121}\frac{\partial^{2}u_{\theta }}{\partial r^{2}}+\frac{A_{2222}}{r^{2}}\frac{\partial^{2}u_{\theta }}{\partial \theta^{2}}+A_{2323}\frac{\partial^{2}u_{\theta }}{\partial z^{2}}\\ \;\;\;\;+\frac{A_{2112}+A_{2211}}{r}\frac{\partial^{2}u_{r}}{\partial r\;\partial \theta }+\frac{A_{2233}+A_{2332}}{r}\frac{\partial^{2}u_{z}}{\partial \theta \;\partial z}\\ \;\;\;\;+\frac{A_{2222}+A_{1212}}{r^{2}}\frac{\partial u_{r}}{\partial \theta }\\ \;\;\;\;+\frac{A_{2121}+A_{1221}-A_{2112}}{r}\frac{\partial u_{\theta }}{\partial r}-\frac{A_{1212}}{r^{2}}u_{\theta } \end{array}$$

(5.8c) $$\begin{array}{l} \rho^{0}\frac{\partial^{2}u_{z}}{\partial t^{2}}=A_{3131}\frac{\partial^{2}u_{z}}{\partial r^{2}}+\frac{A_{3232}}{r^{2}}\frac{\partial^{2}u_{z}}{\partial \theta^{2}}+A_{3333}\frac{\partial^{2}u_{z}}{\partial z^{2}}\\ \;\;\;\;+\left(A_{3113}+A_{3311}\right)\frac{\partial^{2}u_{r}}{\partial r\;\partial z}+\frac{A_{3223}+A_{3322}}{r}\frac{\partial^{2}u_{\theta }}{\partial \theta \;\partial z}\\ \;\;\;\;+\frac{A_{3322}+A_{3113}}{r}\frac{\partial u_{r}}{\partial z}+\frac{A_{3131}}{r}\frac{\partial u_{z}}{\partial r} \end{array}$$

For the initial configuration, the coefficient tensor $B_{IJKL}$ is major-symmetric and only 15 independent components remain which are listed in the Appendix A. Substituting into Equation (4.35), we obtain:

(5.9a) $$\begin{array}{l} \rho^{i}\frac{\partial^{2}U_{r}}{\partial t^{2}}=B_{1111}\frac{\partial^{2}U_{r}}{\partial r^{2}}+\frac{B_{1212}}{r^{2}}\frac{\partial^{2}U_{r}}{\partial \theta^{2}}+B_{1313}\frac{\partial^{2}U_{r}}{\partial z^{2}}\\ \;\;\;\;\;+\frac{B_{1122}+B_{1221}}{r}\frac{\partial^{2}U_{\theta }}{\partial r\;\partial \theta }+\left(B_{1331}+B_{1133}\right)\frac{\partial^{2}U_{z}}{\partial r\;\partial z}\\ \;\;\;\;\;+\frac{B_{1111}}{r}\frac{\partial U_{r}}{\partial r}-\frac{B_{1212}+B_{2222}}{r^{2}}\frac{\partial U_{\theta }}{\partial \theta }\\ \;\;\;\;\;+\frac{B_{1133}-B_{2233}}{r}\frac{\partial U_{z}}{\partial z}-\frac{B_{2222}}{r^{2}}U_{r} \end{array}$$

(5.9b) $$\begin{array}{l} \rho^{i}\frac{\partial^{2}U_{\theta }}{\partial t^{2}}=B_{2121}\frac{\partial^{2}U_{\theta }}{\partial r^{2}}+\frac{B_{2222}}{r^{2}}\frac{\partial^{2}U_{\theta }}{\partial \theta^{2}}+B_{2323}\frac{\partial^{2}U_{\theta }}{\partial z^{2}}\\ \;\;\;\;+\frac{B_{1122}+B_{1221}}{r}\frac{\partial^{2}U_{r}}{\partial r\;\partial \theta }+\frac{B_{2233}+B_{2332}}{r}\frac{\partial^{2}U_{z}}{\partial \theta \;\partial z}\\ \;\;\;\;+\frac{B_{2222}+B_{1212}}{r^{2}}\frac{\partial U_{r}}{\partial \theta }+\frac{B_{2121}}{r}\frac{\partial U_{\theta }}{\partial r}-\frac{B_{1212}}{r^{2}}U_{\theta } \end{array}$$

(5.9c) $$\begin{array}{l} \rho^{i}\frac{\partial^{2}U_{z}}{\partial t^{2}}=B_{3131}\frac{\partial^{2}U_{z}}{\partial r^{2}}+\frac{B_{3232}}{r^{2}}\frac{\partial^{2}U_{z}}{\partial \theta^{2}}+B_{3333}\frac{\partial^{2}U_{z}}{\partial z^{2}}\\ \;\;\;\;+\left(B_{1133}+B_{1331}\right)\frac{\partial^{2}U_{r}}{\partial r\;\partial z}+\frac{B_{2233}+B_{2332}}{r}\frac{\partial^{2}U_{\theta }}{\partial \theta \;\partial z}\\ \;\;\;\;+\frac{B_{1331}+B_{2233}}{r}\frac{\partial U_{r}}{\partial z}+\frac{B_{3131}}{r}\frac{\partial U_{z}}{\partial r} \end{array}$$

Equation (5.8) and Equation (5.9) constitute the acoustoelastic formulations in cylindrical coordinates attached to natural and initial configuration, respectively.

For practical applications, the governing equations given in Equations (5.8) and (5.9) provide the basis for determining wave propagation characteristics in cylindrical structures. The coefficients represent stress-dependent effective elastic moduli. Under appropriate assumptions on the wave field, such as harmonic propagation, these equations can be reduced to characteristic equations for specific wave modes, from which the corresponding wave velocities can be determined. In this sense, the present formulation provides the direct theoretical route from initial stress and material constants to stress-dependent wave velocity in cylindrical structures, which is the key quantity used in acoustoelastic stress evaluation.

### 5.4. Physical Interpretation of Curvature Effects

The acoustoelastic equations derived in cylindrical coordinates differ from their Cartesian counterparts due to the presence of non-zero Christoffel symbols associated with the curvilinear coordinate system. These geometric terms arise from the spatial variation in the basis vectors and represent the intrinsic curvature of the coordinate lines.

From a physical perspective, the additional terms introduced in the cylindrical formulation reflect the influence of geometric curvature on elastic wave propagation. In particular, the coupling between displacement gradients and Christoffel symbols leads to direction-dependent modifications of the effective elastic response. As a result, wave propagation in cylindrical structures is inherently anisotropic, even for materials that are isotropic in their natural configuration.

This curvature-induced effect alters the relationship between stress and wave velocity. Compared to the Cartesian formulation, the cylindrical equations introduce additional contributions to the acoustoelastic coefficient tensor, which modifies the effective stiffness experienced by propagating waves. Consequently, both phase velocity and group velocity may deviate from those predicted under planar assumptions.

The influence of curvature becomes particularly significant when the characteristic radius of the structure is comparable to the wavelength of the propagating wave, or when high-precision measurements are required. In such cases, neglecting curvature effects may lead to systematic errors in the estimation of stress-dependent wave speeds. Therefore, the cylindrical formulation provides a more accurate physical description of wave propagation in curved media and forms an essential extension of classical acoustoelastic theory.

### 5.5. Implications for Ultrasonic Sensing in Cylindrical Structures

In practical ultrasonic sensing applications, cylindrical geometries are widely encountered in engineering structures such as pipelines, pressure vessels, boreholes, and cylindrical composite components. Ultrasonic sensors, including piezoelectric transducers and surface wave probes, are often deployed on curved surfaces to measure stress through wave propagation characteristics.

The acoustoelastic formulation developed in cylindrical coordinates provides a direct link between the stress state in such structures and measurable quantities obtained from ultrasonic sensors. Specifically, the effective elasticity tensor derived in the present framework governs the propagation velocity of elastic waves, which determines measurable parameters such as time-of-flight, phase velocity, and dispersion behavior.

In conventional approaches, wave propagation is frequently approximated using Cartesian formulations, even when applied to curved geometries. However, this approximation neglects curvature-induced effects, which can introduce systematic deviations in the measured wave velocity and consequently affect the accuracy of stress evaluation. The present cylindrical formulation explicitly incorporates these geometric effects, enabling more accurate modeling of wave propagation in curved structures.

Furthermore, the framework is applicable to anisotropic materials, including orthotropic composites commonly used in engineering practice. When the principal material axes do not coincide with the cylindrical coordinate directions, tensor transformations introduce additional coupling terms in the governing equations. These couplings may lead to mode conversion and direction-dependent wave behavior, which are critical factors in ultrasonic sensing and signal interpretation.

From a sensing perspective, the proposed formulation can serve as a forward model that relates internal stress fields to measurable ultrasonic signals. This provides a theoretical basis for improving the accuracy of stress reconstruction algorithms in nondestructive testing and structural health monitoring. Typical applications include pipeline inspection, borehole stress evaluation, and integrity assessment of cylindrical pressure vessels and composite structures.

Overall, the cylindrical acoustoelastic formulation enhances the capability of ultrasonic sensing systems by accounting for geometric curvature and material anisotropy, thereby improving the reliability and precision of stress measurements in practical engineering environments.

## 6. Conclusions

The main conclusions of this work can be summarized as follows:

(a) A general acoustoelastic formulation has been developed within the framework of small-on-large theory. The governing equations are derived in a fully tensorial form, making the formulation independent of any specific coordinate system or material symmetry assumptions. This coordinate-free representation clarifies the intrinsic structure of acoustoelasticity and enables consistent specialization to arbitrary curvilinear coordinate systems through standard tensor transformations.

(b) The general formulation has been explicitly expanded in cylindrical coordinates for orthotropic materials, which are representative of many engineering structures such as rods, pipes, pressure vessels, and boreholes. Compared to conventional Cartesian-based approaches, the present formulation explicitly incorporates geometric curvature through metric and connection terms, leading to modified acoustoelastic coefficients and wave velocities. These effects are particularly relevant for surface and guided waves propagating on curved structures.

(c) From a sensing perspective, the proposed formulation establishes a direct relationship between the stress state and measurable ultrasonic wave characteristics, including wave velocity, time-of-flight, and phase behavior. This provides a rigorous theoretical basis for ultrasonic stress sensing and nondestructive evaluation, particularly in curved and anisotropic structures where conventional planar approximations may introduce systematic errors.

(d) The proposed framework is general and extensible, accommodating arbitrary material symmetry, initial stress states, and coordinate systems within a unified theoretical structure. This makes it suitable for a wide range of sensing applications, including pipeline inspection, borehole stress evaluation, and structural health monitoring of cylindrical components.

(e) The main limitation of the present formulation lies in the increased mathematical complexity associated with curvilinear coordinates and higher-order tensor operations, which may require additional computational effort in practical implementation. Future work will focus on quantitative validation through numerical simulations and experimental measurements, as well as the integration of the present model into ultrasonic sensing and signal interpretation frameworks.

Overall, this study provides a rigorous and flexible theoretical foundation for acoustoelastic-based ultrasonic sensing, extending classical theory beyond Cartesian assumptions and enabling more accurate stress evaluation in curved and anisotropic engineering structures.

## Data Availability

The original contributions presented in this study are included in the article. Further inquiries can be directed to the corresponding author.

## Appendix A

1. Expressions of $A_{\alpha \beta \gamma \delta }$ components for Equation (5.8).

$$\begin{array}{l} A_{1111}=C_{11}+2C_{11}{e^{i}}_{\mathrm{rr}}+D_{111}{e^{i}}_{\mathrm{rr}}+D_{112}{e^{i}}_{\theta \theta }+D_{113}{e^{i}}_{\mathrm{zz}}+{\sigma^{i}}_{\mathrm{rr}}\\ A_{2222}=C_{22}+2C_{22}{e^{i}}_{\theta \theta }+D_{122}{e^{i}}_{\mathrm{rr}}+D_{222}{e^{i}}_{\theta \theta }+D_{223}{e^{i}}_{\mathrm{zz}}+{\sigma^{i}}_{\theta \theta }\\ A_{3333}=C_{33}+2C_{33}{e^{i}}_{\mathrm{zz}}+D_{133}{e^{i}}_{\mathrm{rr}}+D_{233}{e^{i}}_{\theta \theta }+D_{333}{e^{i}}_{\mathrm{zz}}+{\sigma^{i}}_{\mathrm{zz}} \end{array}$$

$$\begin{array}{l} A_{1122}=C_{12}+C_{12}{e^{i}}_{\theta \theta }+C_{66}{e^{i}}_{\theta \theta }+D_{112}{e^{i}}_{\mathrm{rr}}+D_{122}{e^{i}}_{\theta \theta }+D_{123}{e^{i}}_{\mathrm{zz}}\\ A_{2233}=C_{23}+C_{23}{e^{i}}_{\mathrm{zz}}+C_{44}{e^{i}}_{\mathrm{zz}}+D_{123}{e^{i}}_{\mathrm{rr}}+D_{223}{e^{i}}_{\theta \theta }+D_{233}{e^{i}}_{\mathrm{zz}}\\ A_{3311}=C_{31}+C_{31}{e^{i}}_{\mathrm{rr}}+C_{55}{e^{i}}_{\mathrm{rr}}+D_{113}{e^{i}}_{\mathrm{rr}}+D_{123}{e^{i}}_{\theta \theta }+D_{133}{e^{i}}_{\mathrm{zz}} \end{array}$$

$$\begin{array}{l} A_{2211}=C_{12}+C_{12}{e^{i}}_{\mathrm{rr}}+C_{66}{e^{i}}_{\mathrm{rr}}+D_{112}{e^{i}}_{\mathrm{rr}}+D_{122}{e^{i}}_{\theta \theta }+D_{123}{e^{i}}_{\mathrm{zz}}\\ A_{3322}=C_{23}+C_{23}{e^{i}}_{\theta \theta }+C_{44}{e^{i}}_{\theta \theta }+D_{123}{e^{i}}_{\mathrm{rr}}+D_{223}{e^{i}}_{\theta \theta }+D_{233}{e^{i}}_{\mathrm{zz}}\\ A_{1133}=C_{31}+C_{31}{e^{i}}_{\mathrm{zz}}+C_{55}{e^{i}}_{\mathrm{zz}}+D_{113}{e^{i}}_{\mathrm{rr}}+D_{123}{e^{i}}_{\theta \theta }+D_{133}{e^{i}}_{\mathrm{zz}} \end{array}$$

$$\begin{array}{l} A_{2323}=C_{44}+C_{44}{e^{i}}_{\mathrm{zz}}+C_{44}{e^{i}}_{\theta \theta }+D_{144}{e^{i}}_{\mathrm{rr}}+D_{244}{e^{i}}_{\theta \theta }+D_{344}{e^{i}}_{\mathrm{zz}}+{\sigma^{i}}_{\mathrm{zz}}\\ A_{3131}=C_{55}+C_{55}{e^{i}}_{\mathrm{rr}}+C_{55}{e^{i}}_{\mathrm{zz}}+D_{155}{e^{i}}_{\mathrm{rr}}+D_{255}{e^{i}}_{\theta \theta }+D_{355}{e^{i}}_{\mathrm{zz}}+{\sigma^{i}}_{\mathrm{rr}}\\ A_{1212}=C_{66}+C_{66}{e^{i}}_{\mathrm{rr}}+C_{66}{e^{i}}_{\theta \theta }+D_{166}{e^{i}}_{\mathrm{rr}}+D_{266}{e^{i}}_{\theta \theta }+D_{366}{e^{i}}_{\mathrm{zz}}+{\sigma^{i}}_{\theta \theta } \end{array}$$

$$\begin{array}{l} A_{2332}=C_{44}+C_{44}{e^{i}}_{\mathrm{zz}}+C_{23}{e^{i}}_{\theta \theta }+D_{144}{e^{i}}_{\mathrm{rr}}+D_{244}{e^{i}}_{\theta \theta }+D_{344}{e^{i}}_{\mathrm{zz}}\\ A_{1331}=C_{55}+C_{55}{e^{i}}_{\mathrm{zz}}+C_{31}{e^{i}}_{\mathrm{rr}}+D_{155}{e^{i}}_{\mathrm{rr}}+D_{255}{e^{i}}_{\theta \theta }+D_{355}{e^{i}}_{\mathrm{zz}}\\ A_{1221}=C_{66}+C_{66}{e^{i}}_{\theta \theta }+C_{12}{e^{i}}_{\mathrm{rr}}+D_{166}{e^{i}}_{\mathrm{rr}}+D_{266}{e^{i}}_{\theta \theta }+D_{366}{e^{i}}_{\mathrm{zz}} \end{array}$$

$$\begin{array}{l} A_{3232}=C_{44}+C_{44}{e^{i}}_{\theta \theta }+C_{44}{e^{i}}_{\mathrm{zz}}+D_{144}{e^{i}}_{\mathrm{rr}}+D_{244}{e^{i}}_{\theta \theta }+D_{344}{e^{i}}_{\mathrm{zz}}+{\sigma^{i}}_{\theta \theta }\\ A_{1313}=C_{55}+C_{55}{e^{i}}_{\mathrm{rr}}+C_{55}{e^{i}}_{\mathrm{zz}}+D_{155}{e^{i}}_{\mathrm{rr}}+D_{255}{e^{i}}_{\theta \theta }+D_{355}{e^{i}}_{\mathrm{zz}}+{\sigma^{i}}_{\mathrm{zz}}\\ A_{2121}=C_{66}+C_{66}{e^{i}}_{\mathrm{rr}}+C_{66}{e^{i}}_{\theta \theta }+D_{166}{e^{i}}_{\mathrm{rr}}+D_{266}{e^{i}}_{\theta \theta }+D_{366}{e^{i}}_{\mathrm{zz}}+{\sigma^{i}}_{\mathrm{rr}} \end{array}$$

$$\begin{array}{l} A_{3223}=C_{44}+C_{44}{e^{i}}_{\theta \theta }+C_{23}{e^{i}}_{\mathrm{zz}}+D_{144}{e^{i}}_{\mathrm{rr}}+D_{244}{e^{i}}_{\theta \theta }+D_{344}{e^{i}}_{\mathrm{zz}}\\ A_{3113}=C_{55}+C_{55}{e^{i}}_{\mathrm{rr}}+C_{31}{e^{i}}_{\mathrm{zz}}+D_{155}{e^{i}}_{\mathrm{rr}}+D_{255}{e^{i}}_{\theta \theta }+D_{355}{e^{i}}_{\mathrm{zz}}\\ A_{2112}=C_{66}+C_{66}{e^{i}}_{\mathrm{rr}}+C_{12}{e^{i}}_{\theta \theta }+D_{166}{e^{i}}_{\mathrm{rr}}+D_{266}{e^{i}}_{\theta \theta }+D_{366}{e^{i}}_{\mathrm{zz}} \end{array}$$

$${e^{i}}_{\mathrm{rr}}=\frac{\partial {u^{i}}_{r}}{\partial r},\;{e^{i}}_{\theta \theta }=\frac{\partial {u^{i}}_{\theta }}{r\partial \theta }+\frac{{u^{i}}_{r}}{r},$$

2. Expressions of $B_{IJKL}$ components for Equation (5.9).

$$B_{1111}=C_{11}-C_{11}{e^{i}}_{\theta \theta }+3C_{11}{e^{i}}_{\mathrm{rr}}-C_{11}{e^{i}}_{\mathrm{zz}}+D_{112}{e^{i}}_{\theta \theta }+D_{111}{e^{i}}_{\mathrm{rr}}+D_{113}{e^{i}}_{\mathrm{zz}}+{\sigma^{i}}_{\mathrm{rr}}$$

$$B_{2222}=C_{22}+3C_{22}{e^{i}}_{\theta \theta }-C_{22}{e^{i}}_{\mathrm{rr}}-C_{22}{e^{i}}_{\mathrm{zz}}+D_{222}{e^{i}}_{\theta \theta }+D_{122}{e^{i}}_{\mathrm{rr}}+D_{223}{e^{i}}_{\mathrm{zz}}+{\sigma^{i}}_{\theta \theta }$$

$$B_{3333}=C_{33}-C_{33}{e^{i}}_{\theta \theta }-C_{33}{e^{i}}_{\mathrm{rr}}+3C_{33}{e^{i}}_{\mathrm{zz}}+D_{233}{e^{i}}_{\theta \theta }+D_{133}{e^{i}}_{\mathrm{rr}}+D_{333}{e^{i}}_{\mathrm{zz}}+{\sigma^{i}}_{\mathrm{zz}}$$

$$B_{1122}=C_{12}+C_{12}{e^{i}}_{\theta \theta }+C_{12}{e^{i}}_{\mathrm{rr}}-C_{12}{e^{i}}_{\mathrm{zz}}+D_{122}{e^{i}}_{\theta \theta }+D_{112}{e^{i}}_{\mathrm{rr}}+D_{123}{e^{i}}_{\mathrm{zz}}$$

$$B_{2233}=C_{23}+C_{23}{e^{i}}_{\theta \theta }-C_{23}{e^{i}}_{\mathrm{rr}}+C_{23}{e^{i}}_{\mathrm{zz}}+D_{223}{e^{i}}_{\theta \theta }+D_{123}{e^{i}}_{\mathrm{rr}}+D_{233}{e^{i}}_{\mathrm{zz}}$$

$$B_{3311}=C_{31}-C_{31}{e^{i}}_{\theta \theta }+C_{31}{e^{i}}_{\mathrm{rr}}+C_{31}{e^{i}}_{\mathrm{zz}}+D_{123}{e^{i}}_{\theta \theta }+D_{113}{e^{i}}_{\mathrm{rr}}+D_{133}{e^{i}}_{\mathrm{zz}}$$

$$B_{2323}=C_{44}+C_{44}{e^{i}}_{\theta \theta }-C_{44}{e^{i}}_{\mathrm{rr}}+C_{44}{e^{i}}_{\mathrm{zz}}+D_{244}{e^{i}}_{\theta \theta }+D_{144}{e^{i}}_{\mathrm{rr}}+D_{344}{e^{i}}_{\mathrm{zz}}+{\sigma^{i}}_{\mathrm{zz}}$$

$$B_{1313}=C_{55}-C_{55}{e^{i}}_{\theta \theta }+C_{55}{e^{i}}_{\mathrm{rr}}+C_{55}{e^{i}}_{\mathrm{zz}}+D_{255}{e^{i}}_{\theta \theta }+D_{155}{e^{i}}_{\mathrm{rr}}+D_{355}{e^{i}}_{\mathrm{zz}}+{\sigma^{i}}_{\mathrm{zz}}$$

$$B_{1212}=C_{66}+C_{66}{e^{i}}_{\theta \theta }+C_{66}{e^{i}}_{\mathrm{rr}}-C_{66}{e^{i}}_{\mathrm{zz}}+D_{266}{e^{i}}_{\theta \theta }+D_{166}{e^{i}}_{\mathrm{rr}}+D_{366}e_{\mathrm{zz}}+{\sigma^{i}}_{\theta \theta }$$

$$B_{2332}=B_{2323}-{\sigma^{i}}_{\mathrm{zz}};B_{1331}=B_{1313}-{\sigma^{i}}_{\mathrm{zz}};B_{1221}=B_{1212}-{\sigma^{i}}_{\theta \theta }$$

$${e^{i}}_{\mathrm{rr}}=\frac{\partial {U^{i}}_{r}}{\partial r},\;{e^{i}}_{\theta \theta }=\frac{\partial {U^{i}}_{\theta }}{r\partial \theta }+\frac{{U^{i}}_{r}}{r},\;{e^{i}}_{\mathrm{zz}}=\frac{\partial {U^{i}}_{z}}{\partial z}$$

## References

[1] Toupin R.A., Bernstein B. (1961). Sound waves in deformed perfectly elastic materials. Acoustoelastic effect. J. Acoust. Soc. Am..

[2] Thurston R.N., Brugger K. (1964). Third-order elastic constants and the velocity of small amplitude elastic waves in homogeneously stressed media. Phys. Rev..

[3] Pao Y., Gamer U. (1985). Acoustoelastic waves in orthotropic media. J. Acoust. Soc. Am..

[4] Guo J., Fu H., Pan B., Kang R. (2021). Recent progress of residual stress measurement methods: A review. Chin. J. Aeronaut..

[5] Liu Q.H., Sinha B.K. (2003). A 3D cylindrical PML/FDTD method for elastic waves in fluid-filled pressurized boreholes in triaxially stressed formations. Geophysics.

[6] Jasiński R. (2020). Identification of stress states in compressed masonry walls using a non-destructive technique (NDT). Materials.

[7] Jasiński R., Stebel K., Kielan P. (2021). Use of the AE effect to determine the stresses state in AAC masonry walls under compression. Materials.

[8] Maciolek A., Wagener R., Melz T. (2021). Review of and a new approach to elastic modulus evaluation for fatigue design of metallic components. Int. J. Fatigue.

[9] Cauchy A.L. (2009). Sur l’équilibre et le mouvement intérieur des corps considérés comme des masses continues. Oeuvres Complètes; Série 2.

[10] Hughes D.S., Kelly J.L. (1953). Second-Order elastic deformation of solids. Phys. Rev..

[11] Pao Y., Sachse W., Fukuoka H. (1984). Acoustoelasticity and Ultrasonic Measurements of Residual Stresses. Phys. Acoust..

[12] Pao Y. (1987). Theory of acoustoelasticity and acoustoplasticity. Solid Mechanics Research for Quantitative Non-Destructive Evaluation.

[13] Abiza Z., Destrade M., Ogden R.W. (2012). Large acoustoelastic effect. Wave Motion.

[14] Ogden R. (1997). Non-Linear Elastic Deformations, Vol. 1.

[15] Shams M., Destrade M., Ogden R.W. (2011). Initial stresses in elastic solids: Constitutive laws and acoustoelasticity. Wave Motion.

[16] Destrade M., Ogden R.W. (2013). On stress-dependent elastic moduli and wave speeds. IMA J. Appl. Math..

[17] Tang T., Li J., Chen J., Xu Z., Zhang Y. (2024). Investigating acoustoelasticity of plane elastic waves and second harmonics within isotropic solid media: A novel approach. J. Sound Vib..

[18] Tian J., Ma H., Wang M. (2022). A new acoustoelastic model for velocity-hydrostatic-pressure in rocks based on general acoustoelastic theory for elastic solids. SN Appl. Sci..

[19] Kube C.M., Norris A.N. (2022). Stress formulation of acoustoelasticity. Wave Motion.

[20] Kube C.M., Norris A.N. (2021). Stress formulation of elastic wave motion. JASA Express Lett..

[21] de Oliveira D.M.G., Gonçalves V.V., dos Santos Junior A.A. (2024). Modeling the effect of microscale residual stresses on acoustoelasticity for carbon fiber composites. J. Braz. Soc. Mech. Sci. Eng..

[22] Pereira P., Santos A.A. (2013). Influence of Anisotropy Generated by Rolling on the Stress Measurement by Ultrasound in 7050 T7451 Aluminum. Exp. Mech..

[23] Wang W., Zhang Y., Zhou Y., Meng S., Chen D. (2019). Plane stress measurement of orthotropic materials using critically refracted longitudinal waves. Ultrasonics.

[24] Castellano A., Fraddosio A., Marzano S., Daniele Piccioni M. (2017). Some advancements in the ultrasonic evaluation of initial stress states by the analysis of the acoustoelastic effect. Procedia Eng..

[25] Man C.S., Lu W.Y. (1987). Towards an acoustoelastic theory for measurement of residual stress. J. Elast..

[26] Biot M.A. (1939). Non-linear Theory of Elasticity and the linearized case for a body under initial stress. Lond. Edinb. Dublin Philos. Mag. J. Sci..

[27] Biot M.A. (1940). The influence of initial stress on elastic waves. J. Appl. Phys..

[28] Auld B.A. (1973). Acoustic Waveguides. Acoust fields waves solids. Acoust. Fields Waves Solids.

[29] Viktorov I. (1967). Rayleigh waves on Cylinderical and Spherical Surfaces. Rayleigh and Lamb Waves: Physical Theory and Applications.

[30] Hoger A. (1986). On the determination of residual stress in an elastic body. J. Elast..

[31] Haupt P., Pao Y.H., Hutter K. (1992). Theory of incremental motion in a body with initial elasto-plastic deformation. J. Elast..

[32] Holzapfel G.A. (2000). The Concept of Stress. Nonlinear Solid Mechanics: A Continuum Approach for Engineering.

[33] Murdoch I.A. (2012). Calculus in Euclidean Point Space. Physical Foundations of Continuum Mechanics.

[34] Reddy J.N. (2013). Kinematics of continua. An Introduction to Continuum Mechanics.

[35] Surana K.S. (2015). Definitions and measures of stresses. Advanced Mechanics of Continua.

[36] Clifford Truesdell W.N. (2004). The Non-Linear Field Theories of Mechanics.

[37] Tverdokhlebov A. (1983). On the acoustoelastic effect. J. Acoust. Soc. Am..

[38] Dimitrienko Y.I. (2002). Tensor Analysis. Tensor Analysis and Nonlinear Tensor Functions.

[39] Duquennoy M., Ouaftouh M., Ourak M., Xu W. (1999). Influence of natural and initial acoustoelastic coefficients on residual stress evaluation: Theory and experiment. J. Appl. Phys..

[40] Eringen A.C., Robert E. (1980). Tensor analysis. Mechanics of Continua.

[41] Nayfeh A.H. (1995). Material symmetry. Wave Propagation in Layered Anisotropic Media.

[42] Norris A.N. (1991). Symmetry conditions for third order elastic moduli and implications in nonlinear wave theory. J. Elast..

